# Irgm1 Improves Postinfarction Cardiac Repair by Promoting Neutrophil Clearance and Efferocytosis

**DOI:** 10.1002/advs.202514863

**Published:** 2026-02-25

**Authors:** Zeng Wang, Lai Wei, Mingyang Wang, Shanjie Wang, Lili Xiu, Jiaxiang Sun, Rongzhe Lu, Yige Liu, Jiaxin Wang, Fengyi Liu, Weike Liu, Bo Yu, Yong Sun, Xueqin Gao, Shaohong Fang

**Affiliations:** ^1^ The Key Laboratory of Myocardial Ischemia, Chinese Ministry of Education Department of Cardiology, The Second Affiliated Hospital of Harbin Medical University Harbin China; ^2^ State Key Laboratory of Frigid Zone Cardiovascular Disease Harbin Medical University Harbin Heilongjiang China; ^3^ Department of Cardiothoracic Surgery The Sixth Affiliated Hospital of Harbin Medical University Harbin China; ^4^ Department of Integrated Chinese and Western Medicine The First Affiliated Hospital of Harbin Medical University Harbin China

**Keywords:** efferocytosis, IRGM/Irgm1, myocardial infarction, neutrophil clearance, PtdSer exposure

## Abstract

Delayed neutrophil clearance after myocardial infarction (MI) significantly disrupts the myocardial microenvironment, but the underlying mechanisms remain unclear. Macrophage‐mediated efferocytosis of infiltrating neutrophils is crucial for resolving inflammation and restoring homeostasis post‐MI. However, the specific regulatory mechanisms governing neutrophil clearance and efferocytosis remain undefined. This study demonstrates a significant correlation between increased IRGM expression in peripheral blood neutrophils of patients with MI and improved prognostic outcomes. Neutrophil‐specific deletion of Irgm1 exacerbates cardiac dysfunction, impairs post‐MI repair, and hinders neutrophil clearance and efferocytosis. Irgm1 deficiency further delays neutrophil clearance in the heart and extends neutrophil survival. Mechanistically, Irgm1 directly interacts with PDIA3, promoting its autophagic degradation, which in turn activates the endoplasmic reticulum stress/NF‐κB/caspase‐3 pathway to facilitate neutrophil clearance and efferocytosis. In vivo administration of LOC14 significantly reduces tissue damage and enhances cardiac recovery in neutrophil Irgm1‐deficient mice post‐MI. These findings highlight the pivotal role of the Irgm1‐PDIA3 axis in facilitating cardiac repair post‐MI by promoting neutrophil clearance. LOC14 may serve as a potential therapeutic agent to enhance cardiac function post‐MI, particularly in Irgm1‐deficient cases.

## Graphical abstract

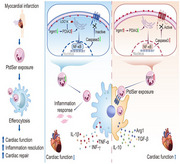

## 1 Introduction

Neutrophil count is established as an independent prognostic marker in patients with acute coronary syndrome (ACS) [1, 2]. Prolonged or intensified neutrophil presence in the heart exacerbates adverse cardiac remodeling, contributing to infarct zone expansion and the acceleration of heart failure (HF) development [3, 4, 5]. Thus, timely reduction of neutrophil accumulation in the heart is essential for promoting optimal scar formation and preventing detrimental remodeling. Experimental anti‐neutrophil strategies—such as inhibiting granulopoiesis in the bone marrow [6], blocking neutrophil recruitment [7, 8], and suppressing neutrophil activation [9, 10] —have proven effective in mitigating tissue damage. However, their clinical translation has been largely unsuccessful. Therefore, identifying the mechanisms underlying neutrophil overaccumulation post‐myocardial infarction (MI) and developing more effective strategies to alleviate the neutrophil burden in infarcted tissues are imperative.

Neutrophils have a half‐life of 6 to 8 h, with their lifespan in circulation typically less than 24 h in mice. However, during inflammatory responses in tissues, this lifespan can extend significantly, ranging from 2 to 4 days [11]. In the post‐MI inflammatory microenvironment, cardiac‐infiltrating neutrophils can prolong their lifespan by delaying apoptosis, resulting in delayed clearance and excessive accumulation [12, 13, 14, 15]. The mechanisms underlying this prolonged neutrophil lifespan remain unclear. Such overaccumulation is detrimental to the myocardial microenvironment, as inadequate neutrophil clearance from the infarct zone can sustain inflammation and exacerbate tissue damage [16, 17]. Therefore, enhancing neutrophil clearance presents a promising strategy to reduce neutrophil burden and improve cardiac function following MI [18].

Neutrophils in tissue are primarily cleared through macrophage‐mediated efferocytosis [17], a process wherein macrophages engulf apoptotic neutrophils [19]. Exposure of phosphatidylserine (PtdSer) on the cell surface is a key hallmark of early apoptosis, acting as a critical “eat me” signal for efferocytosis [20, 21]. Neutrophil apoptosis with PtdSer exposure is efficiently recognized and cleared by macrophages, thus facilitating the resolution of inflammation, rather than late apoptosis and/or necrotic neutrophils [22]. Conversely, delayed PtdSer exposure or its inhibition can result in prolonged apoptosis or inflammatory cell death, such as secondary necroptosis, contributing to persistent inflammation [23, 24]. Therefore, enhancing efferocytosis by modulating neutrophil PtdSer exposure could improve neutrophil clearance and promote resolution of cardiac inflammation in the infarcted heart [25]. However, the specific molecules within neutrophils that regulate PtdSer exposure and efferocytosis following MI remain largely unidentified.

IRGM, a human immune‐related GTPase with a mouse ortholog, Irgm1, belongs to the interferon‐inducible (IFN‐inducible) cytoplasmic immunity‐related GTPase (IRG) family [26, 27]. This family is implicated in various biological processes, including immune responses and immunoregulation. While research on Irgm1 has predominantly focused on infectious and autoimmune diseases [28, 29], its role in cardiovascular diseases, particularly in cardiac remodeling post‐MI, remains underexplored [30], as does its influence on neutrophil function.

In this study, neutrophil‐specific Irgm1 deficiency impaired neutrophil apoptosis and efferocytosis through the PDIA3/ER stress/NF‐κB/caspase‐3 pathway, hindering neutrophil clearance and exacerbating cardiac dysfunction and post‐MI repair. In conclusion, our findings reveal a novel role for the neutrophil Irgm1‐PDIA3 axis in regulating neutrophil clearance and efferocytosis during cardiac repair post‐MI. Targeting this axis may offer a promising therapeutic strategy for HF after MI.

## 2 Result

### 2.1 Upregulated IRGM Level Correlates With Improved Prognostic Indicators Post‐MI

Our previous research identified a correlation between serum IRGM levels and atherosclerotic plaque rupture in patients with acute MI (AMI) [30]. However, its role in ischemic heart injury remains largely undefined. Notably, analysis of IRGM RNA expression across various human cell types using the Human Protein Atlas (www.proteinatlas.org) revealed that IRGM expression is most abundant in oocytes, followed by granulocytes, with moderate expression in T cells and lower levels in macrophages, fibroblasts, smooth muscle cells, and cardiomyocytes (Figure S1A).

**FIGURE 1:**
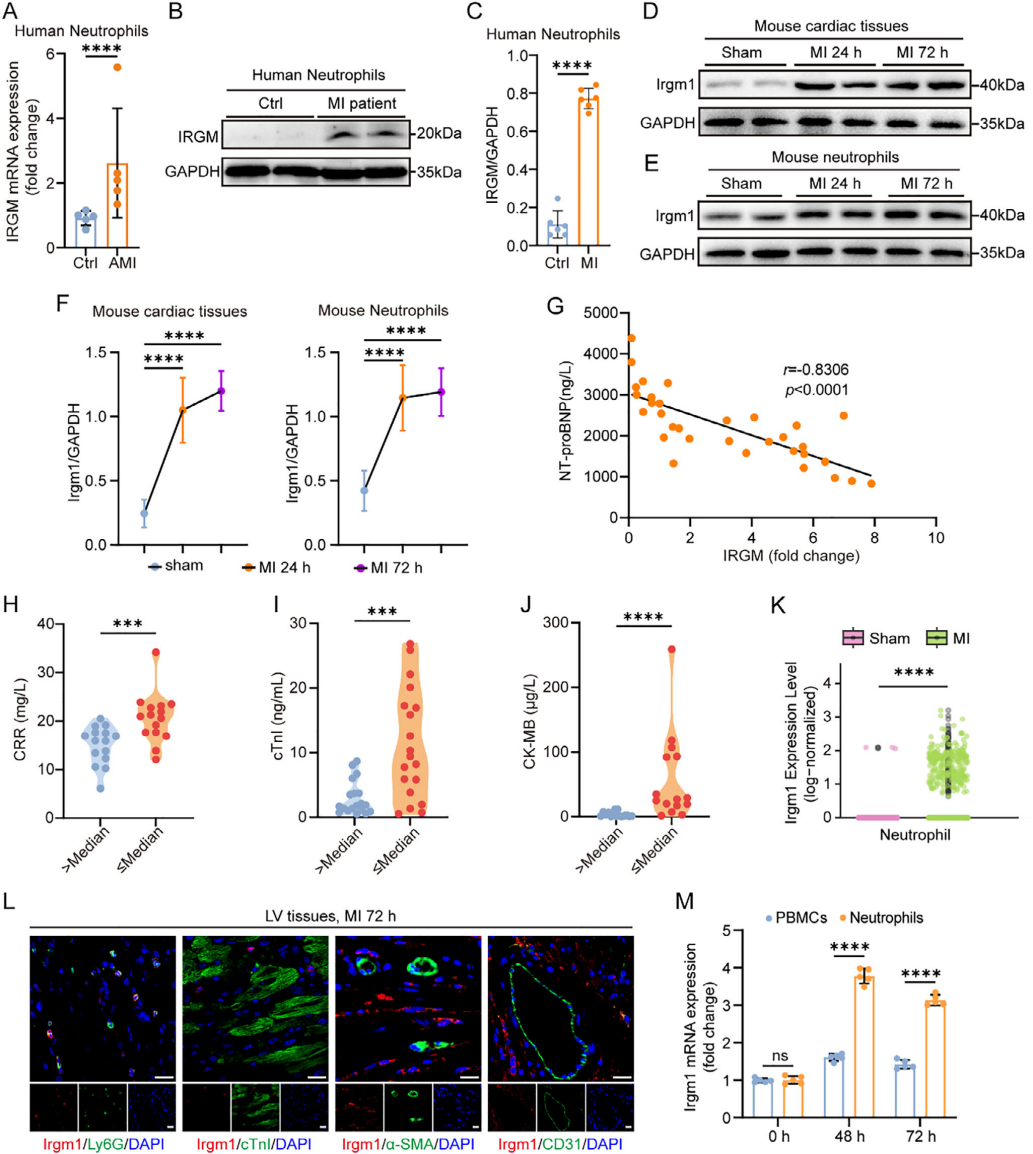
Upregulated IRGM in neutrophils is associated with prognostic indicators post‐MI. (A) Quantitative polymerase chain reaction analysis of IRGM mRNA expression in neutrophils of patients with MI or healthy control (Ctr) individuals (*n* = 5 per group; Mann–Whitney *U*‐test). (B, C) Western‐blot analysis (B) and quantification (C) of IRGM expression in neutrophils from patients with MI or healthy controls (*n* = 6 per group; unpaired Student's *t*‐test). (D, E) Immunoblot analysis of Irgm1 expression in ischemic cardiac tissues (D) or neutrophils (E) of mice after sham operation or indicated days after MI. (F) Quantification of immunoblot band intensity in D (left) and E (right) (*n* = 6 per group; unpaired Student's *t*‐test). (G) Correlation of IRGM mRNA expression in neutrophils with NT‐proBNP levels in the plasma of patients with MI (*n *= 33; Spearman correlation analysis). (H–J) The level of CRP (H) cTnI (I), or CK‐MB (J) in the plasma of patients who have MI with an IRGM level greater than the median compared with patients with an IRGM level less than or equal to the median (*n *= 15 per group for H; *n *= 19 per group for I; *n *= 15 per group for J; Mann–Whitney *U*‐test). (K) Violin plots showing the expression of Irgm1 in cardiac neutrophils. (L) Immunofluorescence staining of Irgm1 in the mouse heart tissues at 72 h post‐MI (Scale bar = 20 µm). (M) The mRNA level of Irgm1 in neutrophils and PBMCs from WT mice after indicated times post‐MI (*n* = 5 per group; unpaired Student's *t*‐test). All data are means ± SD. **p < 0.01, ***p < 0.001, ****p < 0.0001. ns indicates not significant.

Subsequent validation studies confirmed ischemia‐induced upregulation of both IRGM transcripts (Figure 1A) and protein expression (Figure 1B,C) in neutrophils from patients with MI. No significant differences in age, sex, or comorbidities were observed between patients with MI and healthy controls (Table S1). Comparative analysis indicated functional overlap between human IRGM and its mouse counterpart, Irgm1, despite their biochemical differences [31]. Elevated Irgm1 protein levels were also observed in peripheral blood neutrophils and infarct areas of MI mice (Figure 1D–F), suggesting a specific role for IRGM in the pathological mechanisms post‐MI.

To further investigate the role of IRGM in cardiac repair, the relationship between IRGM expression in human neutrophils and key MI prognostic markers—such as N‐terminal pro‐brain natriuretic peptide (NT‐proBNP), cardiac troponin I (cTnI), creatine kinase M‐type (CK‐MB), myoglobin (MB), C‐reactive protein (CRP), interleukin 6 (IL‐6), and interleukin 1β (IL‐1β)—was assessed. These markers are closely associated with MI severity and prognosis [32, 33]. Analysis revealed an inverse correlation between IRGM mRNA expression and circulating NT‐proBNP levels, an established biomarker of HF severity, in patients with MI (Figure 1G). Hepatic and renal dysfunction were excluded based on normal alanine aminotransferase, aspartate aminotransferase, blood urea nitrogen, and creatinine levels (Table S1), ruling out confounding effects on NT‐proBNP levels. Patients were subsequently stratified into “≤ Median” and “> Median” groups based on their IRGM expression levels. The ≤ Median group exhibited significantly higher plasma levels of CRP, IL‐6, and IL‐1β compared to the > Median group (Figure 1H; Figure S1B,C), indicating a link between IRGM expression in neutrophils and exacerbated inflammatory responses after MI. Furthermore, plasma concentrations of cTnI, CK‐MB, and MB were elevated in the ≤ Median group relative to the > Median group (Figure 1I,J; Figure S1D).

The GEO216211 dataset includes gene expression profiles of cardiac neutrophil populations from mice subjected to either sham surgery or MI at day 3 post‐MI. Re‐analysis of this dataset revealed a significant upregulation of Irgm1 expression after MI (Figure 1K). The GSE163129 dataset provides gene expression profiles of cardiac neutrophil populations in mouse hearts one day after either sham surgery or MI. Bioinformatic analysis of this dataset also demonstrated a notable increase in Irgm1 expression in neutrophils at day 1 post‐MI (Figure S1E). Further investigation of Irgm1 expression patterns within the infarcted heart revealed significant co‐localization with the neutrophil marker Ly6G at 72 h post‐MI, with minimal co‐localization with the cardiomyocyte marker cTnI, endothelial marker CD31, or fibroblast marker α‐smooth muscle actin (α‐SMA) (Figure 1L). Irgm1‐positive cells were prominently present in infarcted left ventricular (LV) tissues at days 1, 3, and 7 post‐MI, whereas no or minimal Irgm1 expression was detected in sham‐operated hearts (Figure S1F). These Irgm1‐expressing cells, identified as spherical or ovoid in shape, were localized within damaged myocardial tissues at days 1–3 post‐MI and concentrated in the infarct zone by day 7 (Figure S1F). Further analysis, involving the purification of neutrophils and peripheral blood mononuclear cells (PBMCs) from total peripheral blood leukocytes (PBLs), revealed significantly higher Irgm1 mRNA (Figure 1M) and protein levels (Figure S1G,H) in neutrophils compared to PBMCs at both 24 and 72 h post‐MI. Double immunofluorescence staining confirmed that Irgm1 signals co‐localized with neutrophil markers myeloperoxidase (MPO) and neutrophil elastase (NE) within the infarct zone at 72 h post‐MI (Figure S1I). In vitro experiments showed that stimulation with serum from MI mice significantly upregulated protein levels of Irgm1 in neutrophils (Figure S1J). These results indicate that Irgm1 is upregulated in neutrophils from both peripheral blood and infarcted LV tissue, suggesting a similar expression pattern between human IRGM and mouse Irgm1. As such, neutrophil‐specific Irgm1‐deficient mice provide a reliable animal model for studying human IRGM function. Collectively, these results suggest that upregulated IRGM in neutrophils is strongly associated with reduced cardiac injury and improved HF outcomes post‐MI.

### 2.2 Neutrophil‐Specific Irgm1 Deficiency Aggravated Cardiac Injury and Dysfunction

Given the upregulation of Irgm1 in neutrophils, neutrophil‐specific Irgm1‐deficient mice (Irgm1^flox/flox^‐S100a8‐cre, referred to as Irgm1‐cKO) were generated to investigate the role of Irgm1 in cardiac repair post‐MI (Figure S2A). The baseline characteristics of 8–12 week‐old Irgm1‐cKO and wild‐type (WT) control mice were first evaluated. Genotyping (Figure S2B), qPCR (Figure S2C), and Western blot (Figure S2D,E) confirmed successful deletion of the targeted gene in neutrophils of Irgm1‐cKO mice. Although Irgm1 expression was significantly reduced, it was not completely abrogated in neutrophils, whereas expression remained unchanged in PBMCs of Irgm1‐cKO mice (Figure S2D,E). Blood counts were comparable between Irgm1‐cKO and WT mice (Table S2), and neutrophil morphology was unaffected in Irgm1‐cKO mice (Figure S2F). Additionally, no apparent differences were observed in behavior, body size, spleen, thymus, or body weight between WT and Irgm1‐cKO groups (Figure S2G,H). Baseline cardiac structure and function did not differ significantly between the two groups (Figure S2I–L), and sham‐operated Irgm1‐cKO and WT mice showed no changes in heart size or fibrosis (Figure S2M,N).

**FIGURE 2:**
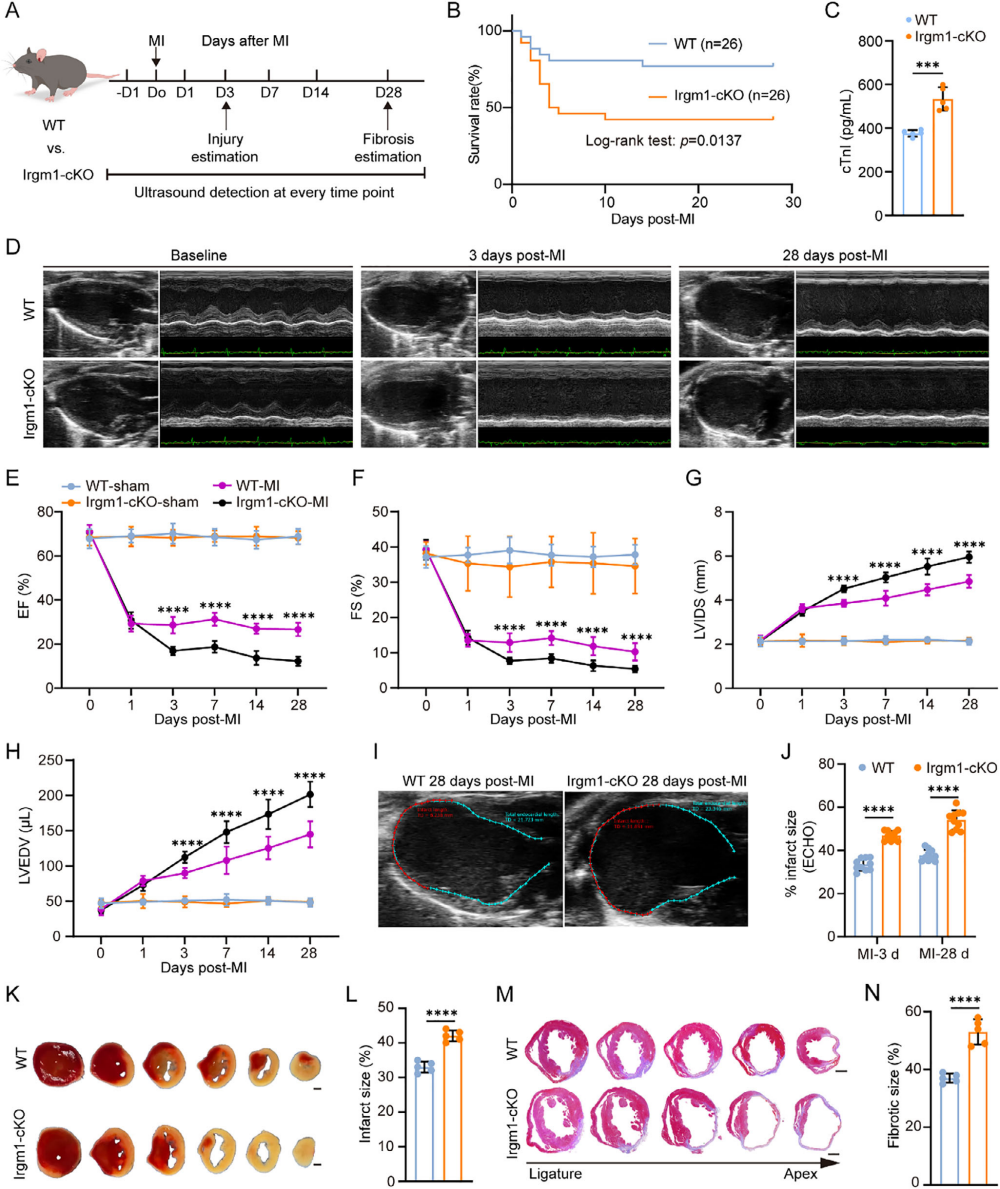
Neutrophil‐specific Irgm1 deficiency exacerbates cardiac dysfunction and promotes adverse cardiac remodeling post‐MI. (A) Schematic diagram of the experimental procedure. (B) The survival rate of WT and Irgm1‐cKO mice within 28 days post‐MI (*n* = 26 per group, Log‐rank test). (C) The level of cardiac troponin I (cTnI) was measured in WT and Irgm1‐cKO mice at 30 hours post‐MI (*n* = 5 per group; unpaired Student's *t‐*test). (D) Representative parasternal long‐axis views and M‐mode echocardiogram images obtained from WT and Irgm1‐cKO mice on days 0, 3, and 28 after MI. (E–H) Echocardiographic analyses of ejection fraction (EF; E), fractional shortening (FS; F), left ventricular internal diameter in systole (LVIDS; G), and left ventricular end‐diastolic volume (LVEDV; H), on days 0, 1,3, 7, 14 and 28 after MI (*n* = 10 per group; 2‐way ANOVA followed by Bonferroni test). (I, J) Post‐MI infarct size was estimated by measuring the length of myocardial infarct (in RED) and total length of LV endocardium (in CYAN) at the middle plane of the long‐axis LV echocardiogram as indicated. Infarct size (%) = (length of infarct/length of LV endocardium) × 100 (*n* = 10 per group; unpaired Student's *t*‐test). (K,L) TTC staining of sequential cardiac sections from WT and Irgm1‐cKO mice on day 3 after MI (K) and the quantified size of infarct area (L) (*n* = 5 per group; unpaired Student's *t*‐test). Scale bar = 1 mm. (M, N) Masson trichrome staining of sequential heart sections from WT and Irgm1‐cKO mice at day 28 after MI (M), and the quantified size of fibrotic areas (N) (*n* = 5 per group; unpaired Student's *t‐*test). Scale bar = 1 mm. All data are means ± SD. ***p < 0.001, ****p < 0.0001.

Post‐MI, the survival rate of Irgm1‐cKO mice at day 28 was lower than that of WT mice (Figure 2A,B). Serum troponin levels in Irgm1‐cKO mice were significantly higher than those in WT mice at 30 h post‐MI (Figure 2C). Furthermore, Irgm1‐cKO mice exhibited worsened cardiac function, as indicated by reduced left ventricular ejection fraction and fractional shortening (Figure 2D–F). In these mice, both the LV internal diameter and LV end‐diastolic volume were increased, signifying more severe LV dilation (Figure 2G,H). On day 1 post‐MI, echocardiography revealed no differences in infarct scar size between Irgm1‐cKO and WT mice. However, by days 3 and 28 post‐MI, the infarct scar size was significantly larger in Irgm1‐cKO mice, likely due to exacerbated LV dilation (Figure 2I,J). Triphenyltetrazolium chloride (TTC) staining at day 3 post‐MI confirmed a larger myocardial infarct area in Irgm1‐cKO mice (Figure 2K,L). Masson's trichrome staining at day 28 post‐MI revealed significantly increased myocardial scar size in Irgm1‐cKO mice (Figure 2M,N). These results demonstrate that Irgm1 deficiency in neutrophils aggravates cardiac dysfunction, intensifies myocardial ischemic injury and fibrosis, and promotes adverse cardiac remodeling after MI.

### 2.3 Neutrophil‐Specific Knockout of Irgm1 Inhibits Cardiac Repair Post‐MI

Efficient cardiac repair after MI is critical for preventing HF. To explore the role of Irgm1 in ischemic cardiac repair, TUNEL staining was performed to assess cardiomyocyte apoptosis. Irgm1‐cKO mice exhibited exacerbated cardiomyocyte apoptosis around the infarct border at day 3 post‐MI (Figure S3A,B). Given that collagen is a major structural component of infarct scar tissue, immunofluorescence staining for fibrotic markers, including collagen III (Col3a1), collagen I (Col1a1), and α‐SMA, was used to evaluate collagen content and maturation post‐MI. On day 7 post‐MI, Irgm1 deficiency led to increased expression of Col3a1 and Col1a1 in the border zone, as well as decreased Col3a1 expression in the infarct zone (Figure S3C‐E,G). However, there were no significant differences in Col1a1 expression in the infarct zone between Irgm1‐cKO and WT mice (Figure S3E,F). Additionally, a marked increase in α‐SMA‐positive profibrotic myofibroblasts was observed (Figure S3H,I), accompanied by a significant reduction in microvessel and capillary density in the infarcted regions of Irgm1‐cKO mice (Figure S3H,J). The expression of profibrotic genes, such as Col3a1, Col1a1, and α‐SMA, was significantly upregulated in Irgm1‐cKO cardiac tissues post‐MI, whereas the mRNA levels of Arginase 1 (Arg1), a marker of the reparative macrophage phenotype, were significantly reduced (Figure S3K–N). These results suggest that Irgm1 deficiency impairs angiogenesis, exacerbates cardiomyocyte apoptosis, and accelerates cardiac fibrosis.

**FIGURE 3:**
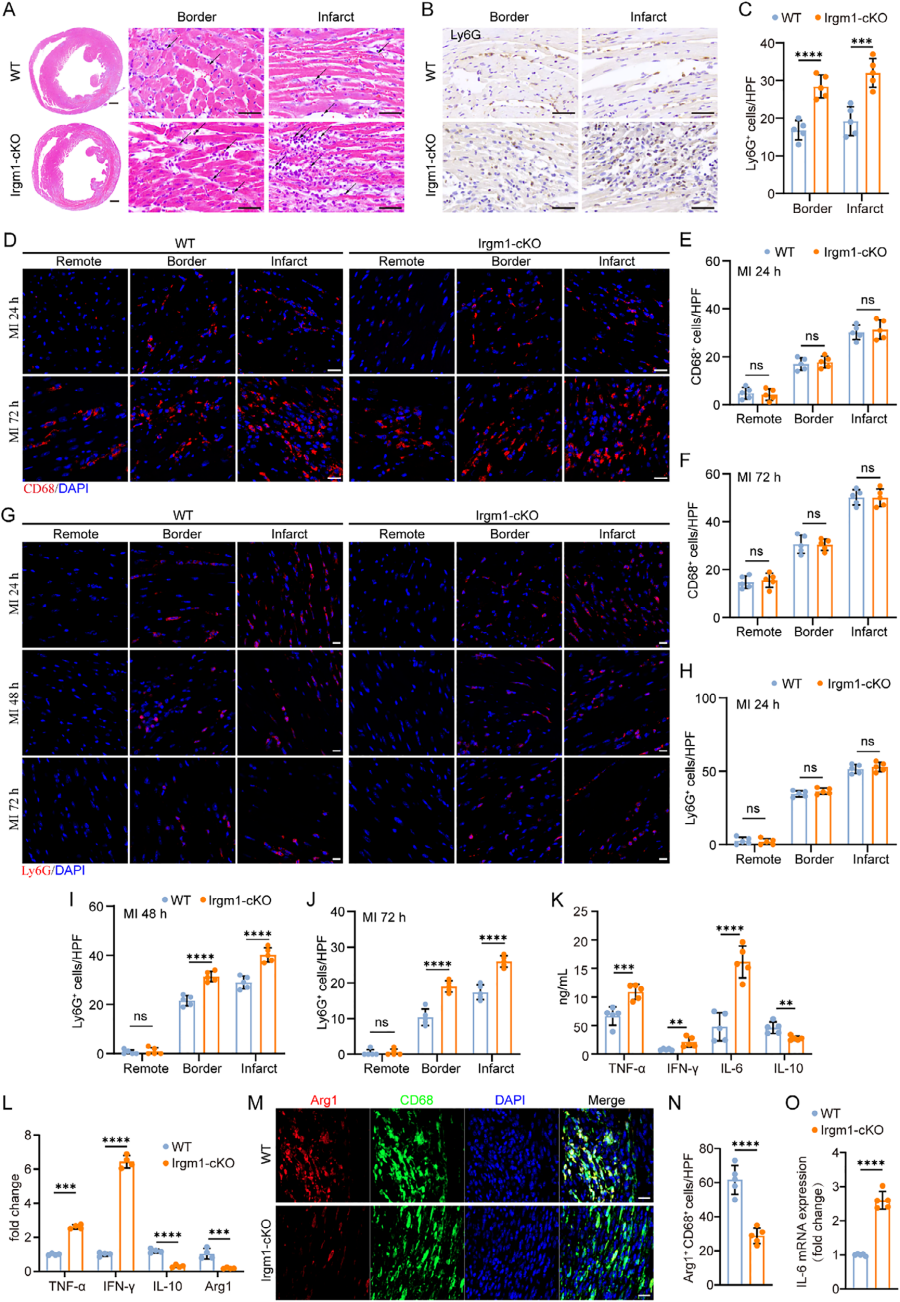
Neutrophil Irgm1‐specific deficiency inhibits the resolution of cardiac inflammation post‐MI. (A) Representative images of H&E staining at day 3 after MI in WT and Irgm1‐cKO mice. Scale bars: 50 µm for high magnification, 500 µm for low magnification. (B, C) Immunohistochemistry staining for Ly6G (B) and quantitative analysis of Ly6G^+^ neutrophils (C) in the infarct zone and border zone of WT and Irgm1‐cKO mice at day 3 post‐MI (*n* = 5 per group; unpaired Student's *t*‐test). Scale bar = 50 µm. (D–F) Immunofluorescence staining for CD68 (D) and quantitative analysis of CD68^+^ macrophages (E, F) in the remote zone, infarct zone, and border zone of WT and Irgm1‐cKO mice at different time points (24 and 72 h) post‐MI (*n *= 5 per group; unpaired Student's *t*‐test). Scale bar = 20 µm. (G–J) Immunofluorescence staining for Ly6G (G) and quantitative analysis of Ly6G^+^ neutrophils (H–J) in the remote zone, infarct zone, and border zone of WT and Irgm1‐cKO mice at different time points (24 h, 48 h, and 72 h) post‐MI (*n* = 5 per group; unpaired Student's *t*‐test and Mann–Whitney *U*‐test). Scale bar = 20 µm. (K) Cytokine levels of tumor necrosis factor‐α (TNF‐α), Interleukin‐6 (IL‐6), interferon‐γ (IFN‐γ), and Interleukin‐10 (IL‐10) in plasma at day 3 post‐MI were detected by Luminex Assays (*n* = 5 per group; unpaired Student's *t*‐test). (L) The mRNA expression levels of proinflammatory genes (TNF‐α and IFN‐γ) and anti‐inflammatory genes (Arg‐1 [arginase‐1] and IL‐10) in cardiac tissue from WT and Irgm1‐cKO at day 3 post‐MI (*n* = 5 per group; unpaired Student's *t‐*test). (M, N) Immunofluorescence staining and quantitative analysis of Arg‐1^+^ macrophages in the cardiac tissues of WT and Irgm1‐cKO mice at day 7 post‐MI (*n* = 5 per group; unpaired Student's *t*‐test). Scale bar = 20 µm. (O) The mRNA expression levels of IL‐6 in ischemic heart tissues of WT and Irgm1‐cKO mice at day 7 post‐MI (*n* = 5 per group; unpaired Student's *t*‐test). All data are means ± SD. ***p < 0.001, ****p < 0.0001. ns indicates not significant.

Timely inflammation resolution post‐MI is essential to prevent progression to HF. To assess the impact of Irgm1 on inflammation resolution, H&E staining revealed higher densities of inflammatory cells in both the infarct border zone and the infarct zone in Irgm1‐cKO mice. Close examination revealed a significant increase in the number of cells with smaller, more densely packed nuclei, characteristic of neutrophils (Figure 3A, indicated by arrows). Immunohistochemistry using the neutrophil marker Ly6G confirmed increased neutrophil infiltration in both the infarct border and infarct zones in Irgm1‐cKO mice compared to WT mice (Figure 3B,C), suggesting that Irgm1 deficiency may influence neutrophil infiltration. Immunofluorescence staining revealed no significant differences in macrophage numbers between the infarct and border zones of the heart in either group at 24 and 72 h post‐MI (Figure 3D–F). At 24 h post‐MI, both WT and Irgm1‐cKO mice showed robust recruitment of Ly6G^+^ neutophils to the infarcted heart, with no significant differences between the groups (Figure 3G,H). However, at 48 and 72 h post‐MI, Irgm1‐cKO mice exhibited significantly higher neutrophil counts in both the infarct and border zones compared to WT mice (Figure 3G,I,J), indicating that Irgm1 deficiency affects neutrophil infiltration in the infarcted heart.

On day 3 post‐MI, proinflammatory cytokines such as tumor necrosis factor‐α (TNF‐α), interferon‐γ (IFN‐γ), and IL‐6 were elevated in Irgm1‐cKO mice, while the anti‐inflammatory cytokine interleukin‐10 (IL‐10) was reduced (Figure 3K). This cytokine imbalance created a proinflammatory and profibrotic environment, with increased TNF‐α and IFN‐γ levels and decreased Arg‐1 and IL‐10 levels (Figure 3L). Additionally, Irgm1 knockout diminished Arg‐1‐positive reparative macrophages within ischemic cardiac tissue (Figure 3M,N), while IL‐6 levels remained elevated on day 7 post‐MI (Figure 3O), suggesting delayed inflammation resolution in Irgm1‐cKO hearts.

### 2.4 Neutrophil‐Specific Irgm1 Deficiency Delays the Clearance of Cardiac Infiltrating Neutrophils Post‐MI

**FIGURE 4:**
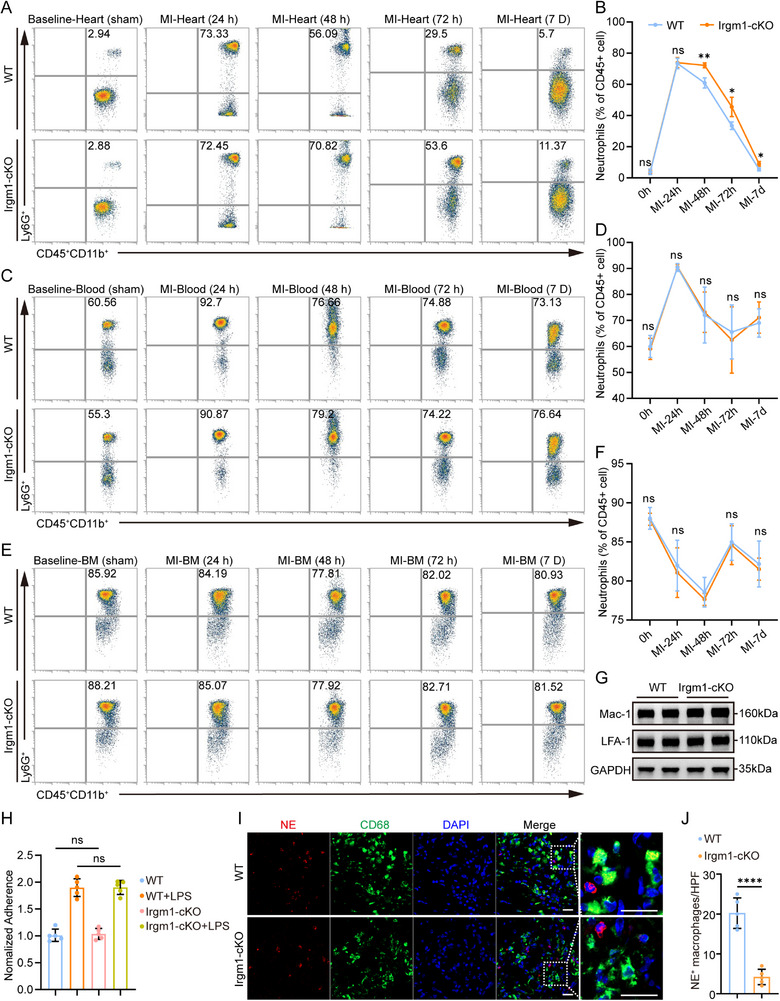
Neutrophil‐specific Irgm1 deficiency results in delayed neutrophil clearance in cardiac post‐MI. (A–F) Flow cytometric analysis of neutrophils (CD45^+^, CD11b^+^, Ly6G^+^) in the heart from WT and Irgm1‐cKO mice at different time points (24 h, 48 h, 72 h, and 7 days) after MI or sham operation (A, C, E), along with their quantification (B, D, F). (*n* = 5 per group; 2‐way ANOVA followed by Bonferroni test). (G) Western blot analysis (H) of LFA‐1 and Mac‐1 expression in blood neutrophils from WT and Irgm1‐cKO mice post‐MI. (H) The static adhesion of WT and Irgm1‐cKO neutrophils to ECs was analyzed, where ECs were pre‐plated as a confluent layer in culture dishes (*n* = 5 per group; one‐way ANOVA followed by Bonferroni test). Neutrophils were 5‐(and ‐6)‐carboxyfluorescein diacetate succinimidyl esters (CFSE)‐labeled (to facilitate counting on ECs) and treated with or without exRNaseA. For assaying adhesion, neutrophils were added to EC‐plated dishes for 15 min, followed by washes to remove unattached or loosely attached neutrophils. Cells were then counted under a microscope, with adherence quantified as the number of neutrophils averaged across at least 10 random imaging fields, and normalized to the control (WT without LPS stimulation) group. Each dot represents a biological replicate. Data from a representative experiment are shown. (I) Immunofluorescence staining shows colocalization of CD68^+^ macrophages with NE^+^ of WT and Irgm1‐cKO mice at day 3 post‐MI. Scale bar = 20 µm. (J) Quantification of the CD68^+^ macrophages with NE^+^ neutrophils in the cardiac tissues of WT and Irgm1‐cKO mice at day 3 post‐MI (*n* = 5 per group; unpaired Student's *t*‐test). All data are means ± SD. *p < 0.05, **p < 0.01, ***p < 0.0001. ns indicates not significant.

The recruitment and infiltration of neutrophils post‐MI were further investigated (Figure S4A–C). A significant increase in neutrophil recruitment to the heart was observed within 24 h post‐MI, peaking during the first 24 h of the 7‐day observation period (Figure 4A,B). A similar trend was noted in blood neutrophil counts, which also peaked at 24 h (Figure 4C,D). Consistently, the number of neutrophils in the bone marrow initially showed a significant decrease, followed by a recovery (Figure 4E,F), which is consistent with previous studies [34], supporting the initial mobilization of neutrophils primarily from the bone marrow, subsequently leading to the generation of new neutrophils. Notably, no significant differences in neutrophil numbers were found in the heart, blood, or bone marrow of Irgm1‐cKO mice 24 h after MI compared to WT mice (Figure 4B,D,F), suggesting that Irgm1 does not influence neutrophil generation or mobilization during AMI. Similarly, no differences were observed in neutrophil numbers in the blood or bone marrow between the two groups at 48 h, 72 h, and 7 days post‐MI (Figure 4D,F). However, a significant difference in cardiac neutrophil numbers was detected at 48 h, 72 h, and 7 days post‐MI (Figure 4B). These results suggest that while Irgm1 deficiency does not affect neutrophil generation, mobilization, and recruitment, it may impair neutrophil clearance from the heart after MI.

The functional recruitment of neutrophils was also assessed. Neutrophil adhesion to the endothelium is a critical step in their recruitment to the heart after MI [35, 36]. White blood cell integrins LFA‐1 (CD11a/CD18) and Mac‐1 (CD11b/CD18) are essential for neutrophil adhesion [37, 38]. Our results indicate that Irgm1 deficiency does not alter the expression of LFA‐1 or Mac‐1 (Figure 4G). To further investigate whether Irgm1 deficiency affects neutrophil adhesion to endothelial cells, the adhesion of LPS‐treated WT and Irgm1‐cKO neutrophils to endothelial cells was quantified. The data indicate that Irgm1 deficiency does not influence neutrophil adhesive function, suggesting that it does not impact neutrophil recruitment (Figure 4H).

Neutrophil clearance is primarily mediated by macrophage efferocytosis [19]. To investigate the role of Irgm1 in this process, neutrophil efferocytosis in Irgm1‐deficient mice was examined. Immunofluorescence staining revealed a significant reduction in neutrophil efferocytosis in Irgm1‐deficient mice compared to WT controls (Figure 4I,J).

In vitro, primary neutrophils from the bone marrow of WT and Irgm1‐cKO mice were labeled with 5(6)‐carboxyfluorescein succinimidyl amino ester (CFSE) after LPS stimulation. CFSE‐labeled neutrophils were subsequently co‐cultured with bone marrow‐derived macrophages (BMDMs) for 1 h (Figure S5A). Immunofluorescence staining showed a marked reduction in both the number of efferocytic macrophages and the efferocytosis index in Irgm1‐deficient samples compared to WT neutrophils (Figure S5B,C). Additionally, Irgm1 deficiency led to increased expression of proinflammatory markers TNF‐α and IFN‐γ, while decreasing anti‐inflammatory markers Arg‐1 and IL‐10 in BMDMs (Figure S5D,E). Supplementing Irgm1‐deficient neutrophils with recombinant Irgm1 protein (R‐Irgm1) promoted efferocytosis (Figure S5F,G) and partially restored the anti‐inflammatory phenotype of BMDMs (Figure S5H–K). To assess whether recombinant Irgm1 protein could enter neutrophils, the recombinant protein was engineered with a His tag and co‐cultured with neutrophils for 2 h. Western blot and immunofluorescence analysis confirmed that the recombinant protein successfully entered the neutrophils (Figure S5L,M). These results suggest that recombinant Irgm1 functions within neutrophils. Irgm1 deficiency impairs neutrophil efferocytosis and prevents the phenotypic transition of macrophages to a reparative state.

Additionally, baseline and post‐MI (at 24 h, 48 h, 72 h, and 7 days) monocyte populations in blood, heart tissue, and bone marrow were comparable between Irgm1‐cKO and WT mice (Figure S6A–F), indicating that Irgm1 deficiency does not alter monocyte/macrophage recruitment or infiltration. In summary, these results suggest that while Irgm1 deficiency does not affect granulopoiesis, neutrophil development, differentiation, or initial recruitment to the infarcted heart, it impairs neutrophil clearance post‐MI.

### 2.5 Neutrophil‐Specific Irgm1 Deficiency Delaying Neutrophil Clearance Through the Caspase3 Pathway

Among the various methods of neutrophil clearance, apoptosis causes the least tissue damage. Apoptotic neutrophils are cleared through efferocytosis, a process that suppresses inflammation and promotes tissue repair [39, 40]. TUNEL staining at day 3 post‐MI revealed significantly lower neutrophil apoptosis in Irgm1‐cKO mice compared to WT mice (Figure S7A), indicating that Irgm1 deficiency impedes neutrophil apoptosis. Flow cytometric analysis (Figure S4D) further confirmed a significant reduction in the percentage of Ly6G^+^A5^+^ neutrophils in Irgm1‐cKO infarcts at day 2 post‐MI, relative to WT mice (Figure S7B,C), suggesting that Irgm1 deficiency inhibits neutrophil apoptosis.

To directly investigate whether Irgm1 is involved in neutrophil apoptosis in vivo, an adoptive transfer experiment was performed (Figure S7D). In vitro‐purified Irgm1‐cKO and WT neutrophils were labeled with CFSE and injected into WT mice with MI. After 24 h, the number of CSFE^+^ neutrophils was assessed by flow cytometry (Figure S4E). Compared to the WT neutrophil transfer group, the number of CFSE^+^ neutrophils was significantly higher in the Irgm1‐cKO neutrophil transfer group (Figure S7E,F), directly demonstrating that the deletion of Irgm1 in neutrophils leads to delayed neutrophil death in vivo. This finding indicates that Irgm1 deficiency enhances neutrophil survival.

**FIGURE 5:**
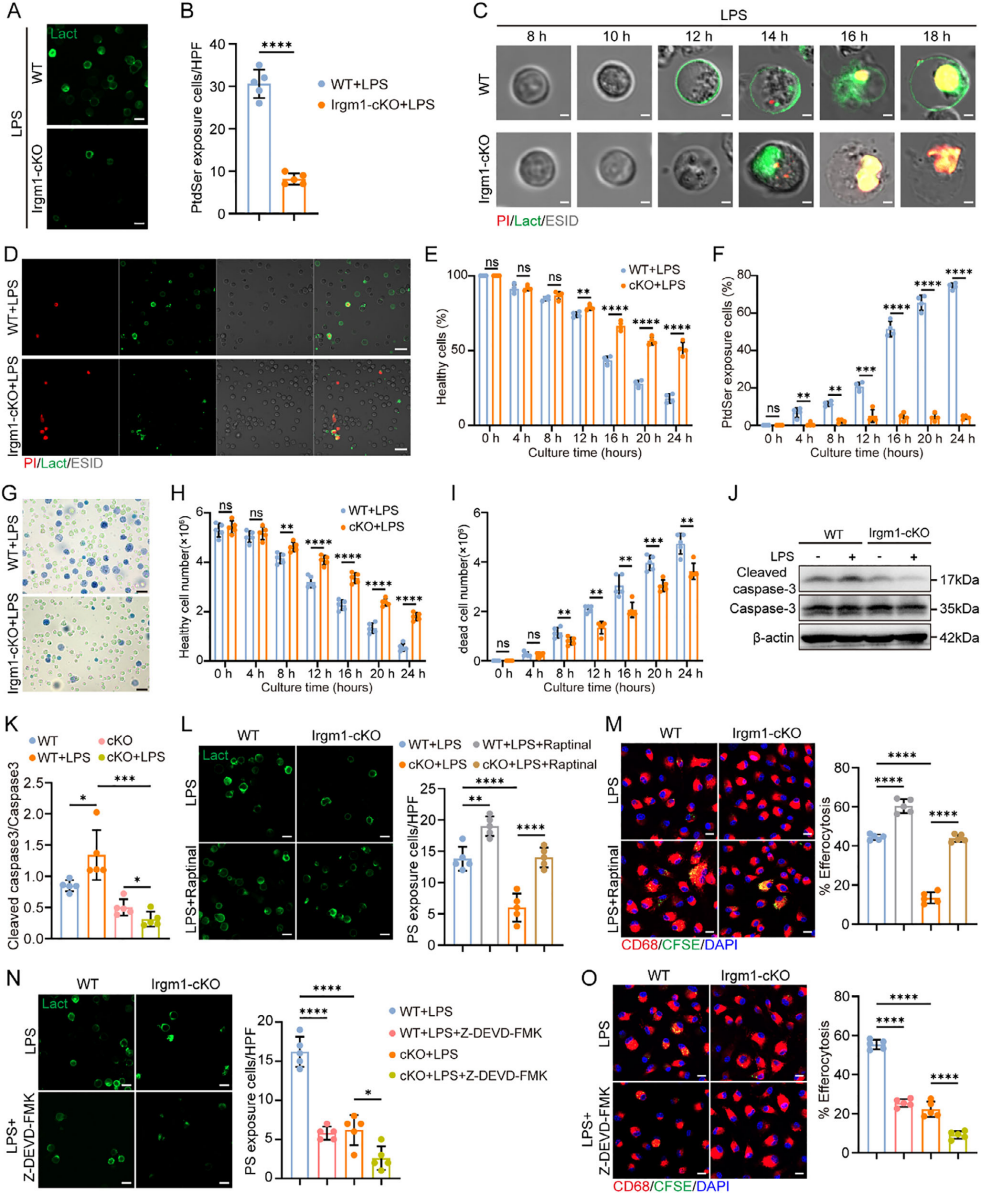
Neutrophil‐specific Irgm1 deficiency inhibits PtdSer exposure and efferocytosis through the caspase‐3 pathway. (A, B) FITC‐conjugated lactadherin (FITC‐Lact) staining of neutrophils with PtdSer exposure after LPS treatment (A) and its quantitative analysis (B). (*n *= 5 per group; unpaired Student's *t‐*test). Scale bar = 10 µm. Neutrophil undergoing PtdSer exposure (early apoptosis) exhibits diffuse rim staining. (C) Representative images of neutrophils with FITC‐Lact and propidium iodide (PI) co‐staining at the indicated time points. Scale bar = 2 µm. (D) Representative images of neutrophils with FITC‐Lact and propidium iodide (PI) co‐staining. Results are representative of at least five independent experiments. Scale bar = 20 µm. (E, F) The percentage of healthy (PI negative and FITC‐Lact‐negative, E), and PtdSer exposure (FITC‐Lact‐positive, F) neutrophils (*n* = 4 per group; unpaired Student's *t*‐test). PtdSer exposure: PtdSer externalized to the cell membrane surface exhibits diffuse rim staining. Healthy cell: PI negative and PtdSer exposure negative. (G) Representative images of neutrophils with trypan blue staining at the indicated time points. Results are representative of at least five independent experiments. Scale bar = 200 µm. (H, I) Absolute counts of healthy neutrophils (H) and dead neutrophils (I). (*n* = 5 per group; unpaired Student's *t*‐test). (J, K) Western blot analysis (J) and quantification (K) of Cleaved caspase‐3 expression in neutrophils treated with LPS (*n* = 5 per group; one‐way ANOVA followed by Bonferroni test). (L) FITC‐conjugated lactadherin (FITC‐Lact) staining and quantification of PtdSer exposure in neutrophils with LPS and Raptinal treatment (*n* = 5 per group; one‐way ANOVA followed by Bonferroni test). Scale bar = 10 µm. (M) Immunofluorescence images showing BMDMs in the process of engulfing neutrophils and the corresponding efferocytosis index (*n* = 5 per group; one‐way ANOVA followed by Bonferroni test). Neutrophils cultured with LPS and Raptinal were subsequently labeled with CFSE. Scale bar = 10 µm. (N) FITC‐conjugated lactadherin (FITC‐Lact) staining and quantification of PtdSer exposure in neutrophils with LPS and Z‐DEVD‐FMK treatment (*n *= 5 per group; one‐way ANOVA followed by Bonferroni test). Scale bar = 10 µm. (O) Immunofluorescence images showing BMDMs in the process of engulfing neutrophils and the corresponding efferocytosis index. Neutrophils cultured with LPS and Z‐DEVD‐FMK were subsequently labeled with CFSE. (*n* = 5 per group; one‐way ANOVA followed by Bonferroni test). Scale bar = 10 µm. All data are means ± SD. *p < 0.05, **p < 0.01, ***p < 0.001, ****p < 0.0001. ns indicates not significant.

The effects of Irgm1 deficiency on neutrophil apoptosis and survival rates were further investigated in vitro. PtdSer exposure (PtdSer externalized to the cell membrane), a marker of early apoptosis, serves as a key “eat‐me” signal for macrophage recognition and phagocytosis [20, 21]. Lactadherin, which binds more specifically to early PtdSer than Annexin V, was used to detect this exposure [41, 42]. Confocal microscopy revealed abundant PtdSer exposure in purified WT neutrophils after 16 h of LPS stimulation, whereas Irgm1‐cKO neutrophils exhibited significantly reduced PtdSer exposure (Figure 5A,B), indicating that Irgm1 deficiency impairs PtdSer exposure. Next, PtdSer exposure was assessed by immunofluorescence analysis of propidium iodide (PI) and Lact co‐staining. In the WT group, most neutrophils exhibited prominent PtdSer exposure (early apoptosis), which subsequently progressed to late apoptosis (PtdSer exposure and PI positive). In Irgm1‐cKO neutrophils, most cells were PI‐positive without evidence of PtdSer exposure (Figure 5C,D), suggesting that Irgm1 deficiency inhibits early neutrophil apoptosis. In addition, the ratio of healthy neutrophils in the Irgm1‐cKO group was significantly increased compared to WT neutrophils (Figure 5D,E), while the PtdSer exposure rate was significantly reduced (Figure 5D,F), suggesting that Irgm1 deficiency inhibited PtdSer exposure and prolonged neutrophil survival. To further examine the impact of Irgm1 on neutrophil survival, the absolute number of neutrophils at each time point was measured using trypan blue staining. Notably, the Irgm1‐cKO group contained significantly more healthy neutrophils compared to the WT group at the same time point (Figure 5G,H), whereas the Irgm1‐cKO group also had significantly fewer dead neutrophils than the WT group (Figure 5G,I). These results suggest that Irgm1 deficiency extends neutrophil survival by inhibiting PtdSer exposure.

The mechanism by which Irgm1 regulates PtdSer exposure was further investigated. Initially, this study examined whether Irgm1 directly binds to PtdSer using proximity ligation assays (PLA). No positive PLA signals were detected between Irgm1 and PtdSer (Figure S8), suggesting that Irgm1 does not directly bind to PtdSer. Given the critical role of the caspase‐3 pathway in regulating PtdSer exposure [43, 44], immunoblot analysis was performed, which revealed markedly reduced cleaved caspase‐3 protein levels in Irgm1‐cKO neutrophils following LPS stimulation (Figure 5J,K). Treatment of Irgm1‐cKO neutrophils with Raptinal, a caspase‐3 activator, partially promoted neutrophil PtdSer exposure (Figure 5L) and efferocytosis (Figure 5M). Conversely, treatment with the caspase‐3 inhibitor Z‐DEVD‐FMK resulted in a significant reduction in neutrophil PtdSer exposure (Figure 5N) and efferocytosis (Figure 5O). In summary, these results demonstrate that Irgm1 deficiency delays neutrophil clearance and extends neutrophil lifespan by inhibiting the caspase‐3 signaling pathway.

### 2.6 Irgm1 Promotes Neutrophil Clearance by Interacting with PDIA3 to Induce its Degradation

**FIGURE 6:**
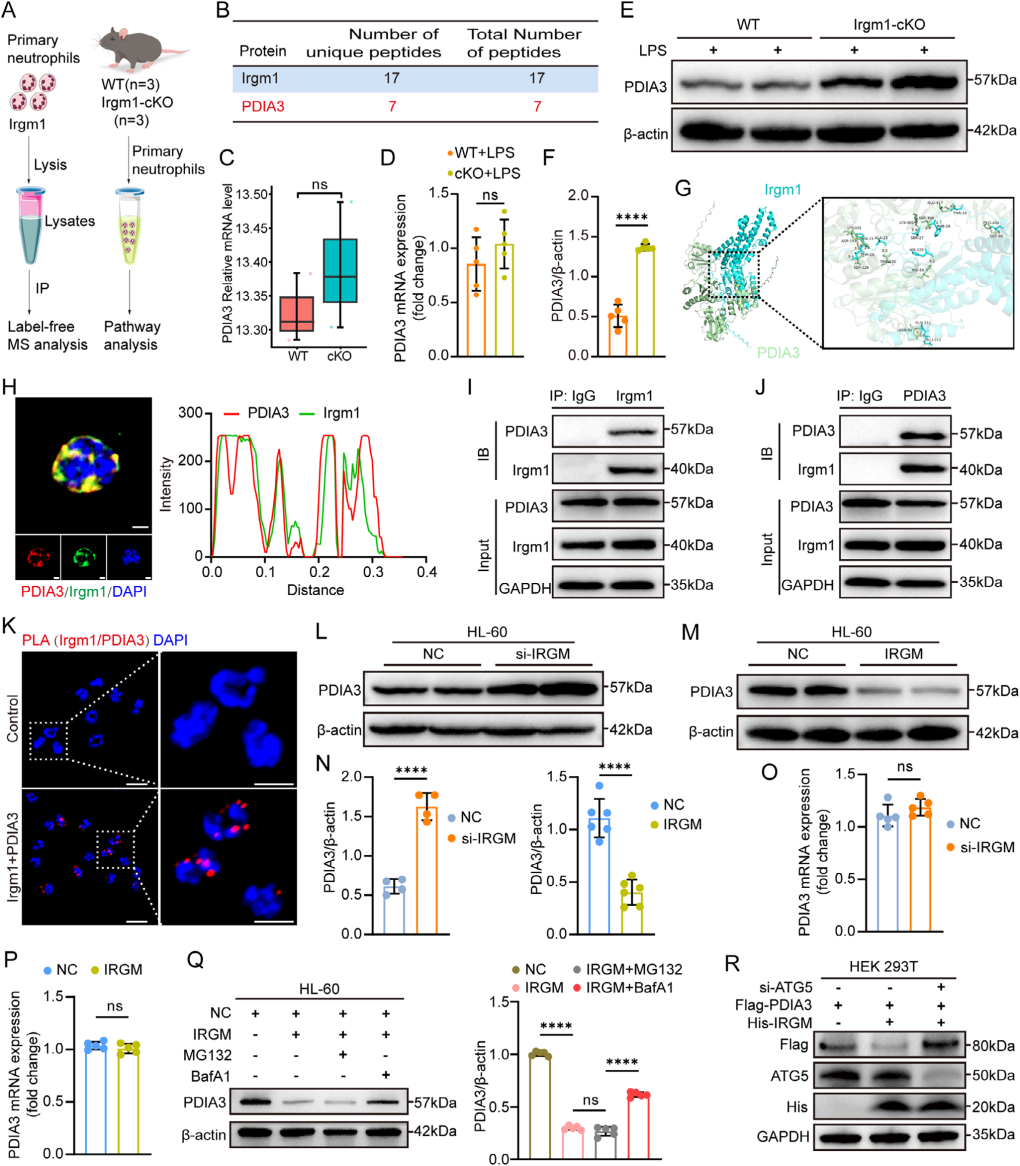
Irgm1 interacts with PDIA3 to mediate its degradation. (A) Schematic diagram of the experimental design for IP–MS analysis (left) and RNA–seq analysis (right). (B) Potential Irgm1‐interacting proteins identified by MS analysis. (C) Boxplot of PDIA3 expression level analysis from RNA–seq analysis (*n *= 3 per group; unpaired Student's *t*‐test). (D) The mRNA expression levels of PDIA3 in WT and Irgm1‐cKO neutrophils treated with LPS (*n *= 5 per group, unpaired Student's *t*‐test). (E and F) Western‐blot analysis (E) and quantification (F) of PDIA3 expression in neutrophils after LPS treatment (*n *= 5 per group; unpaired Student's *t‐*test). (G) Representative image of autodocking between Irgm1 and PDIA3 protein. (H) Representative images of immunofluorescence staining of Irgm1 along with PDIA3 in neutrophils (left), and analyses of the co‐location curve (right). Scale bar = 2 µm. (I) Western blot of PDIA3 level following immunoprecipitation with anti‐Irgm1 antibody in neutrophils. (J) Western blot of Irgm1 level following immunoprecipitation with anti‐PDIA3 antibody in neutrophils. (K) The Signals of protein–protein interactions between Irgm1 and PDIA3 were examined by PLA using NaveniFlexTM Cell MR in situ red starter kit mouse/rabbit (red). Scale bar = 10 µm. (L) Representative bands of PDIA3 expression in HL‐60 cells transfected with si‐IRGM. (M) Representative bands of PDIA3 expression in HL‐60 cells after overexpression of IRGM. (N) Quantification of immunoblot band intensity in L(left) and M (right) (*n* = 4 per group for N and *n* = 6 per group for M; unpaired Student's *t*‐test). (O) The mRNA expression levels of PDIA3 in HL‐60 cells transfected with si‐IRGM (*n* = 5 per group; unpaired Student's *t‐*test). (P) The mRNA expression levels of PDIA3 in HL‐60 cells after overexpression of IRGM (*n *= 5 per group, unpaired Student's *t*‐test). (Q) Effect of IRGM overexpression on PDIA3 protein in the presence of MG132 or Bafilomycin A1 by western blot assay (*n *= 5 per group; one‐way ANOVA followed by Bonferroni test). (R) ATG5‐siRNA transfected HEK293T cells expressing Flag‐PDIA3 and His‐IRGM were analyzed by western blot assay. All data are means ± SD. ****p < 0.0001. ns indicates not significant.

To investigate how Irgm1 regulates PtdSer exposure and efferocytosis in neutrophils, immunoprecipitation Mass Spectrometry (IP‐MS) was performed to identify Irgm1 interacting partners (Figure 6A). The analysis identified protein disulfide‐isomerase A3 (PDIA3) as a potential interacting partner (Figure 6B). Additionally, RNA‐seq of neutrophils treated with LPS from both WT and Irgm1‐cKO mice was conducted to analyze differential signaling pathways (Figure 6A). RNA‐seq analysis and q‐PCR results showed no significant changes in the expression of PDIA3 target genes (Figure 6C,D); however, a notable increase in PDIA3 protein levels was observed (Figure 6E,F). These results suggest that Irgm1 may be involved in the post‐transcriptional regulation of PDIA3.

To explore the interaction between Irgm1 and PDIA3, molecular docking simulations were conducted using Autodock. The results indicated that these proteins could form stable hydrogen bonds through multiple residue groups, suggesting a potential interaction (Figure 6G). Immunofluorescence assays further confirmed the colocalization of Irgm1 and PDIA3 in neutrophils exposed to LPS (Figure 6H). Co‐immunoprecipitation (Co‐IP) analysis was then conducted to investigate whether PDIA3 binds to Irgm1. As expected, PDIA3 strongly co‐immunoprecipitated with Irgm1 in LPS‐exposed neutrophils (Figure 6I,J). Additionally, PLA was performed to visually detect protein–protein interactions using confocal microscopy. As shown in Figure 6K, a significant interaction was observed between Irgm1 and PDIA3 within the neutrophil cytoplasm, confirming a direct interaction between the two proteins. To assess the impact of IRGM on PDIA3 in HL‐60 cells (a neutrophil cell line), knockdown and overexpression experiments were conducted (Figure S9A,B). Knockdown of IRGM led to a significant increase in PDIA3 expression, while overexpression of IRGM resulted in a reduction of PDIA3 levels (Figure 6L–N). Interestingly, modulation of IRGM expression did not affect the mRNA levels of PDIA3 (Figure 6O,P), further indicating that Irgm1 directly interacts with PDIA3 and negatively regulates its protein levels

The mechanism by which Irgm1 regulates PDIA3 protein levels was further investigated. Cycloheximide experiments showed that the absence of Irgm1 significantly prolonged the half‐life of PDIA3 protein, an effect that was reversed by overexpression of Irgm1 (Figure S9C,D). Treatment with the proteasome inhibitor MG132 did not block the degradation of PDIA3 mediated by Irgm1, while the autophagy inhibitor bafilomycin A1 inhibited PDIA3 degradation, suggesting that Irgm1‐mediated PDIA3 degradation depends on autophagy rather than the ubiquitin‐proteasome system (Figure 6Q). Additionally, Irgm1 deficiency suppressed neutrophil autophagy levels (Figure S10A,B). In the absence of the essential autophagy protein ATG5, IRGM was unable to degrade PDIA3 (Figure 6R). Previous studies have demonstrated that Irgm1 promotes p62‐dependent autophagy and subsequent protein degradation. In this study, Irgm1 and PDIA3 were shown to interact with p62 (Figure S10C,D). These findings demonstrate that Irgm1 facilitates PDIA3 degradation via the autophagy pathway.

PDIA3, a member of the protein disulfide isomerase (PDI) family, primarily functions in the folding of newly synthesized glycoproteins and in responding to endoplasmic reticulum (ER) stress [45, 46]. Previous studies have shown that PDIA3 inhibits ER stress‐induced apoptosis [47]. Ultrastructural analysis using transmission electron microscopy (TEM) revealed swollen ER morphology and a shorter, disorganized structure in WT neutrophils treated with LPS, indicating ER stress after LPS stimulation. In contrast, the ER in Irgm1‐cKO neutrophils displayed a disorganized morphology, but swelling was less pronounced, and the overall ER volume was significantly reduced, indicating that Irgm1 deficiency suppressed the level of ER stress in neutrophils (Figure S11A). GRP78, a classical marker of ER stress, showed reduced expression in Irgm1‐cKO neutrophils, as evidenced by Western blot analysis (Figure S11B,C) and decreased fluorescence intensity compared to WT neutrophils (Figure S11D,E). Treatment with LOC14, a PDIA3 inhibitor, partially restored ER stress in LPS‐treated Irgm1‐cKO neutrophils, as observed through TEM (Figure S11F). Consistent with this, LOC14 treatment also upregulated GRP78 expression (Figure S11G,H) and increased fluorescence intensity (Figure S11I,J). Notably, LOC14 treatment contributed to partial recovery of PtdSer exposure (Figure S11K) and efferocytosis (Figure S11L,M) in Irgm1‐cKO neutrophils. These results suggest that Irgm1 regulates ER stress and promotes neutrophil clearance through its interaction with PDIA3.

### 2.7 Irgm1‐PDIA3 Axis Delays Neutrophil Clearance and Efferocytosis via the ER Stress /NF‐κB/Caspase3 Pathway

To investigate the downstream signaling pathways of the Irgm1‐PDIA3 axis, RNA sequencing (RNA‐seq) and pathway analysis of differentially expressed genes (DEGs) were performed in neutrophils, revealing a strong association between NF‐κB signaling and Irgm1 ablation (Figure S12A). Among the four identified pathways, positive regulation of NF‐κB signaling was linked to caspase‐3, as indicated by gene ontology enrichment analysis (Figure S12B). Furthermore, GSEA of the RNA‐seq data showed a significant downregulation of NF‐κB signaling upon Irgm1 deletion (Figure S12C). Previous studies have shown that caspase‐3 acts as an apoptosis‐related protein downstream of the NF‐κB signaling pathway, and the NF‐κB/caspase‐3 pathway is a key signaling axis inducing apoptosis [48]. In addition, ER stress can induce apoptosis through activation of the NF‐κB pathway [49]. Therefore, these results suggest that activation of the ER stress/NF‐κB/caspase‐3 axis may play a critical role in Irgm1‐PDIA3 axis‐mediated PtdSer exposure in neutrophils.

To validate this hypothesis, the expression of P65, a key component of NF‐κB, was examined. Following LPS stimulation, nuclear translocation of P65 was observed in WT neutrophils, but this translocation was markedly attenuated in Irgm1‐cKO neutrophils (Figure S12D). Compared to WT neutrophils, Irgm1‐cKO neutrophils exhibited significantly elevated PDIA3 expression, alongside reduced levels of ER stress markers (GRP78 and ATF6), p‐P65, and cleaved caspase‐3 (Figure S12E,F). To further confirm that Irgm1 functions through the ER stress/NF‐κB/caspase‐3 axis, IRGM overexpression was induced in HL‐60 cells using an IRGM plasmid. The results showed that IRGM overexpression significantly decreased PDIA3 expression while markedly increasing the levels of ER stress markers (GRP78 and ATF6), p‐P65, and cleaved caspase‐3 (Figure S12G,H). Inhibition of ER stress using 4‐PBA reduced the expression levels of GRP78, ATF6, p‐P65, and cleaved caspase‐3 (Figure S12I,J). Concomitantly, 4‐PBA treatment significantly suppressed neutrophil PtdSer exposure and efferocytosis (Figure S12K,L). Collectively, these results demonstrate that the Irgm1‐PDIA3 axis activates the ER stress/NF‐κB/caspase‐3 signaling pathway, thereby promoting neutrophil clearance.

### 2.8 Supplementation With LOC14 Improves Cardiac Repair and Cardiac Function Post‐MI

**FIGURE 7:**
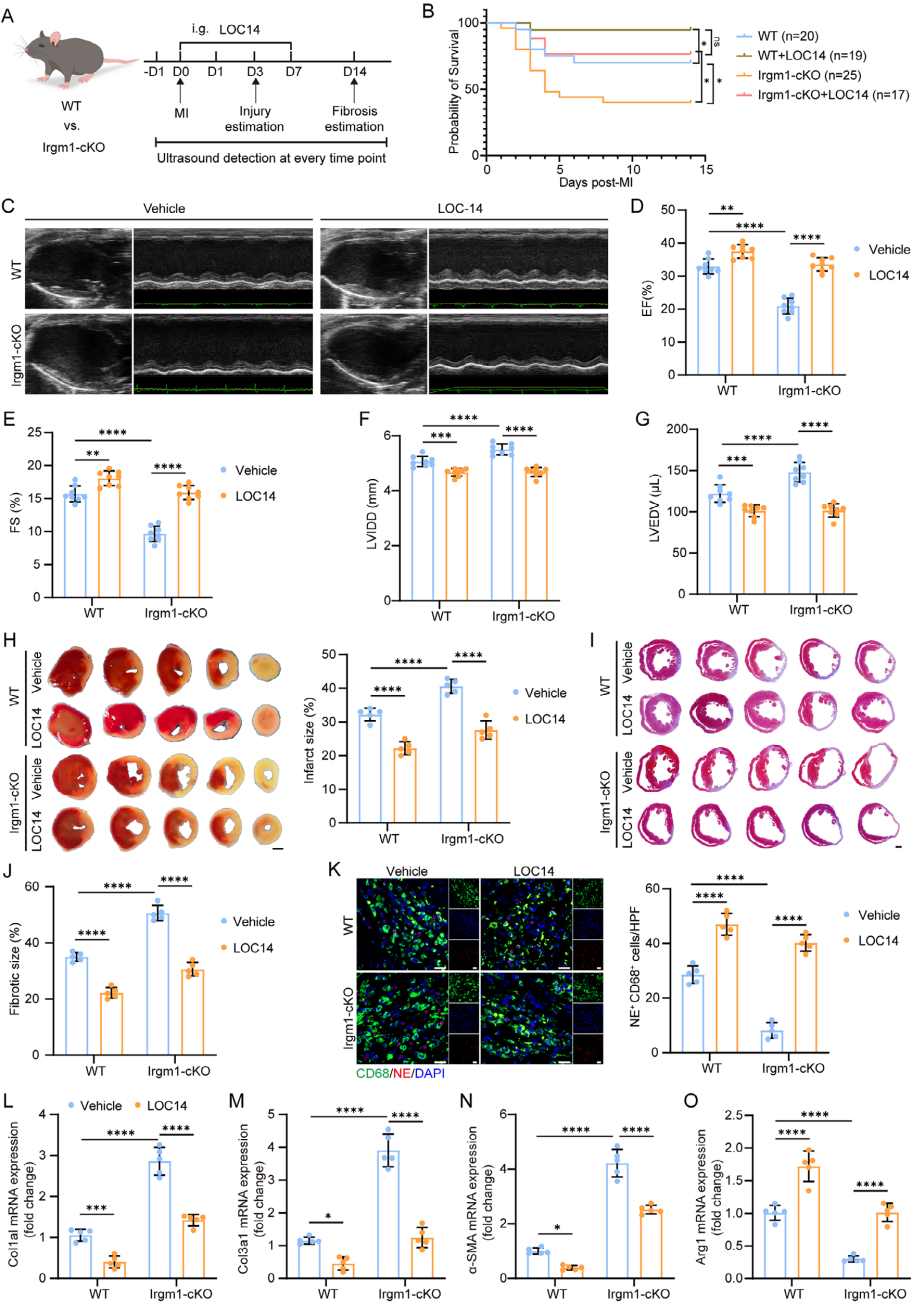
Administration of LOC14 negates the detrimental effects of Irgm1 deficiency after MI. (A) Experimental strategy of the MI model. (B) The survival rate of WT and Irgm1‐cKO mice with or without treatment of LOC14 at day 14 post‐MI (*n *= 20 for vehicle‐treated WT mice, *n *= 19 for LOC14‐treated WT mice, *n *= 25 for vehicle‐treated Irgm1‐cKO mice, *n *= 17 for LOC14‐treated Irgm1‐cKO mice. Log‐rank test). (C) Representative parasternal long‐axis views and M‐mode echocardiogram images obtained from WT and Irgm1‐cKO mice on days 0 and 14 post‐MI. (D–G) Echocardiographic analyses of ejection fraction (EF; D), fractional shortening (FS; E), left ventricular internal diameter in Diastole (LVIDD; F), and left ventricular end‐diastolic volume (LVEDV; G) on days 14 post‐MI (*n* = 8 per group; two‐way ANOVA followed by Bonferroni test). (H) TTC staining of sequential cardiac sections from WT and Irgm1‐cKO mice on day 3 after MI and the quantified size of fibrotic areas (*n* = 5 per group; two‐way ANOVA followed by Bonferroni test). Scale bar = 1 mm. (I) Masson trichrome staining of sequential heart sections from WT and Irgm1‐cKO mice at day 14 after MI (I) and the quantified size of fibrotic areas (J) (*n* = 5 per group; two‐way ANOVA followed by Bonferroni test). Scale bar = 500 µm. (K) Immunofluorescence staining shows colocalization of CD68^+^ macrophages with NE^+^ from WT and Irgm1‐cKO mice at day 3 post‐MI, and quantification of the CD68^+^ macrophages with NE^+^ (*n* = 5 per group; two‐way ANOVA followed by Bonferroni test). Scale bar = 20 µm. (L–O) The mRNA expression levels of genes associated with cardiac fibrosis and myocardial repair in ischemic heart tissues of Irgm1‐cKO mice with or without treatment of LOC14 at day 14 post‐MI (*n* = 5 per group; two‐way ANOVA followed by Bonferroni test). All data are means ± SD. *p < 0.05, **p < 0.01, ***p < 0.001, ****p < 0.0001.

Although the neutrophil Irgm1‐PDIA3 axis plays a critical role after MI, the absence of pharmacological inhibitors targeting Irgm1 limits its therapeutic exploration. Therefore, the potential of pharmacologically targeting PDIA3, a downstream effector of Irgm1, was assessed as a therapeutic approach for MI. LOC14, a PDIA3 inhibitor, has not been explored for its therapeutic effects in MI [50, 51]. LOC14 was administered daily for seven consecutive days to both WT and Irgm1‐cKO mice after MI (Figure 7A). After LOC14 treatment, the level of ER stress in neutrophils of Irgm1‐cKO mice was partially restored (Figure S13A–C). Compared to WT controls, Irgm1‐cKO mice exhibited a decreasing trend in survival rate, although this difference was not statistically significant after LOC14 treatment (Figure 7B). Irgm1‐cKO mice also displayed lower cardiac function than WT mice, as evidenced by reduced ejection fraction and fractional shortening (Figure 7C–E). Both WT and Irgm1‐cKO mice showed significant improvement in cardiac function after LOC14 treatment, with a more pronounced improvement observed in the Irgm1‐cKO group (Figure 7C–E). Furthermore, LOC14 treatment had beneficial effects on cardiac remodeling, significantly reducing LV volume and internal diameter post‐MI (Figure 7F,G).

TTC staining revealed that the infarct size on day 3 post‐MI was larger in Irgm1‐cKO mice compared to WT mice; however, LOC14 treatment significantly reduced the infarct size in both WT and Irgm1‐cKO mice (Figure 7H). Masson's trichrome staining showed that 14 days post‐MI, the fibrotic scar area was greater in Irgm1‐cKO mice; LOC14 treatment reduced the fibrotic scar area in both WT and Irgm1‐cKO hearts (Figure 7I,J). Immunofluorescence staining showed that efferocytosis by neutrophils was significantly reduced in Irgm1‐cKO mice, whereas treatment with LOC14 improved efferocytosis of neutrophils in both WT and Irgm1‐cKO mice in the heart (Figure 7K). Notably, compared to WT mice, Irgm1‐cKO mice exhibited significantly elevated expression of genes associated with cardiac fibrosis and markedly decreased expression of genes associated with macrophage repair phenotype post‐MI (Figure 7L–O). Following LOC14 treatment, the expression of fibrosis‐related genes was partially reduced in both Irgm1‐cKO and WT mice, while the expression of macrophage repair phenotype‐associated genes was enhanced (Figure 7L–O).

**SCHEME 1:**
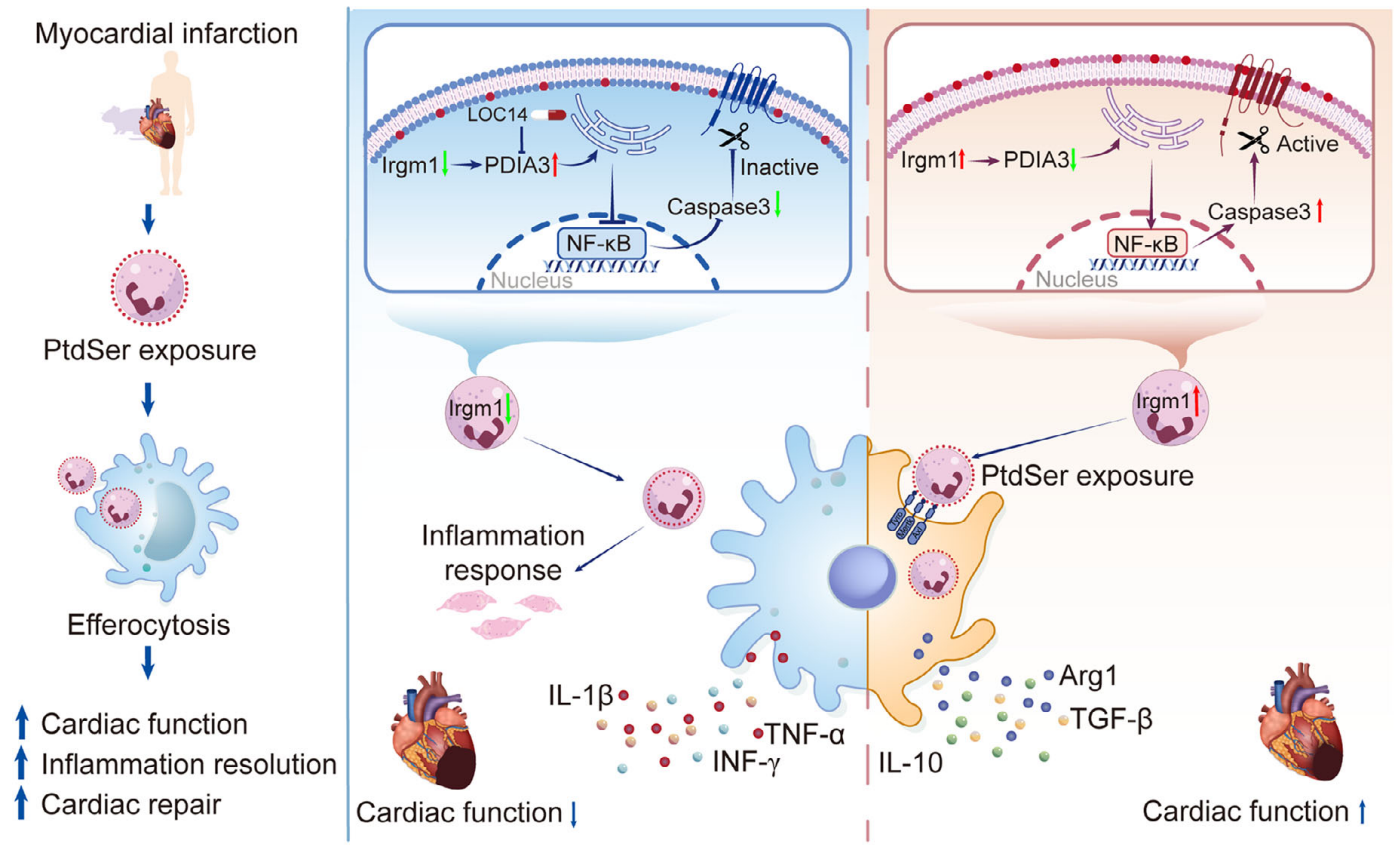
Schematic illustration of the Irgm1‐mediated neutrophil Clearance and macrophage efferocytosis. The Irgm1‐PDIA3 axis enhances post‐infarction cardiac repair by accelerating neutrophil clearance and facilitating efferocytosis. Irgm1 holds potential as a prognostic biomarker in MI, and LOC14 may represent a therapeutic option to improve cardiac repair, especially in cases of Irgm1 deficiency.

Furthermore, to determine whether LOC14 exerts effects specific to the pathological state of MI or non‐specific systemic effects, sham‐operated mice received LOC14 treatment. No significant changes in cardiac function were observed among the groups following treatment (Figure S14A–E). H&E staining and Masson's trichrome staining demonstrated normal myocardial tissue architecture with no inflammatory cell infiltration or fibrosis in all groups (Figure S14F,G). These results suggest that LOC14 holds therapeutic potential in alleviating post‐MI cardiac injury and improving cardiac remodeling by inhibiting PDIA3, particularly in individuals with Irgm1 deficiency (Scheme 1).

## 3 Discussion

Although IRGM/Irgm1 has been implicated in various inflammatory and autoimmune diseases [28, 30, 31], its role in MI and neutrophil function remains underexplored. This study demonstrates that neutrophil‐specific deletion of Irgm1 impairs neutrophil clearance and efferocytosis, contributing to worsened cardiac function post‐MI. Specifically, in the absence of Irgm1, elevated PDIA3 suppresses PtdSer exposure via the ER stress/NF‐κB/caspase‐3 pathway, hindering efferocytosis and neutrophil clearance. Targeting the Irgm1‐PDIA3 axis with LOC14 may offer a promising therapeutic approach for improving cardiac repair and function post‐MI, particularly in individuals with Irgm1 deficiency.

Neutrophil clearance is essential for inflammation resolution after MI, and delayed neutrophil clearance or excessive accumulation leads to myocardial infarct expansion [16, 17]. In vivo, neutrophil‐specific deletion of Irgm1 impaired cardiac tissue repair, exacerbated cardiac dysfunction, and delayed neutrophil clearance in the heart. Neutrophil clearance occurs through cell death pathways, such as apoptosis, necrosis, and pyroptosis [23, 39], with efferocytosis following apoptosis being the most favorable [52]. Our results suggest that Irgm1 deficiency inhibits both neutrophil apoptosis and efferocytosis. The clearance of apoptotic neutrophils initiates inflammation resolution, while their efferocytosis by macrophages triggers the production of anti‐inflammatory mediators [14]. Furthermore, Irgm1 deficiency suppresses the expression of anti‐inflammatory mediators and the reparative phenotype of cardiac macrophages, delaying the heart's transition to inflammation resolution. These findings highlight Irgm1's role in cardiac protection through the regulation of neutrophil clearance and efferocytosis post‐MI.

As terminally differentiated effector cells, neutrophils infiltrating the infarcted myocardium undergo apoptosis within 48 h. This apoptotic process releases chemotactic nucleotides (“find‐me” signals) and induces membrane externalization of PtdSer, the dominant “eat‐me” signal [53], enabling precise recognition and clearance by macrophages. Notably, PtdSer exposure on the outer leaflet is one of the most evolutionarily conserved phagocytic markers [21]. When PtdSer exposure is delayed or suppressed, efferocytosis is inhibited [24, 54]. Irgm1 deficiency reduces PtdSer exposure on neutrophils, thereby inhibiting efferocytosis. Neutrophils can extend their lifespan by delaying apoptosis [55]. Additionally, Irgm1 deficiency inhibits early neutrophil apoptosis, resulting in increased survival and significantly prolonged lifespan. Efferocytosis of neutrophils by macrophages is critical for tissue homeostasis and inflammation resolution post‐MI [19]. Notably, cells in early apoptosis, rather than late apoptosis or necrosis, can induce the expression of tissue repair genes in macrophages [22]. In this study, macrophages that phagocytosed Irgm1‐deficient neutrophils exhibited significantly reduced anti‐inflammatory factors and elevated pro‐inflammatory factors. Additionally, in vitro experiments showed that Irgm1 deficiency reduced early apoptosis and increased necrosis in neutrophils. These results indicate that efferocytosis of Irgm1‐deficient neutrophils fails to effectively trigger the macrophage phenotypic transformation needed for tissue repair.

PDIA3, a chaperone protein, plays a critical role in modulating glycoprotein folding and responding to ER stress [45, 46]. This study is the first to identify that Irgm1 directly interacts with PDIA3, promoting its degradation. Previous research has shown that PDIA3 is involved in the autophagy‐lysosomal pathway [46], while Irgm1 is a key mediator of autophagy and the subsequent degradation of proteins through this process [31]. Our findings indicate that Irgm1 is responsible for the autophagic degradation of PDIA3. Elevated PDIA3 expression has been shown to suppress ER stress and inhibit ER stress‐induced apoptosis [47]. In contrast, our results suggest that Irgm1, by repressing the upregulation of PDIA3, activates ER stress and promotes neutrophil apoptosis, as indicated by increased PtdSer exposure.

Caspase‐3 activation plays a pivotal role in promoting PtdSer exposure on the outer layer of the plasma membrane, which is crucial for the recognition and removal of apoptotic cells [43, 44, 56]. During apoptosis, caspase‐3 activation facilitates PtdSer exposure on the cell surface, leading to the engulfment of apoptotic cells by phagocytes [25]. Our results indicate that the Irgm1‐PDIA3 axis enhances neutrophil PtdSer exposure and efferocytosis by facilitating caspase‐3 activation. Caspase‐3, a pro‐apoptotic protein downstream of the NF‐κB signaling pathway, is involved in apoptosis induction, as highlighted in previous research [48]. ER stress can induce apoptosis through the activation of NF‐κB [57]. Furthermore, in our previous study, we found that in atherosclerosis, IRGM/Irgm1 regulates reactive oxygen species (ROS) production and phosphorylation of JNK/p38/ERK in the MAPK signaling pathway to influence macrophage apoptosis [30]. In this study on neutrophils, we revealed a novel mechanism: Irgm1 promotes the degradation of PDIA3, thereby triggering the ER stress/NF‐κB/Caspase‐3 pathway, which ultimately facilitates PtdSer exposure (early apoptosis) and efferocytosis. Given the expression of Irgm1 in macrophages and neutrophils and its role in different disease models, this brings new insights. We will continue to study the function and mechanism of macrophage Irgm1 in subsequent projects post‐MI.

LOC14, a PDIA3 inhibitor, has demonstrated anti‐influenza and anti‐pulmonary fibrosis effects through targeted inhibition of PDIA3 activity [50, 51]. Our initial findings indicate that LOC14 can effectively promote cardiac function recovery post‐MI, particularly in mice lacking neutrophil‐specific Irgm1. This suggests that LOC14, as a targeted inhibitor of the Irgm1‐PDIA3 axis, may have significant clinical potential for treating patients with MI. Of course, future studies will need to employ more specific approaches, such as conditional knockdown of PDIA3 specifically in neutrophils, to ultimately confirm its independent role post‐MI.

However, this study has several limitations. First, while this study confirmed that Irgm1 promotes neutrophil PtdSer exposure and efferocytosis by regulating the autophagic degradation of PDIA3, other proteins may also be involved in this process. Second, there is currently no specific agonist for Irgm1, and developing one could be crucial for future clinical applications. Third, considering the timing of neutrophil clearance after MI, the most effective window for drug intervention may be early in the post‐MI period. Fourth, targeting PDIA3 locally in the heart through gene therapy combined with targeted delivery systems could offer additional therapeutic potential. Finally, due to the low transfection efficiency in neutrophils, achieving effective overexpression of Irgm1 both in vitro and in vivo remains challenging. Future studies should aim to establish neutrophil‐specific Irgm1 overexpression mouse models, which are critical for further investigating the functional roles of Irgm1 in neutrophils.

## 4 Conclusion

In conclusion, this study highlights the critical role of Irgm1 in post‐MI tissue repair and the maintenance of cardiac function, primarily through regulating neutrophil clearance and efferocytosis. These findings unveil a novel pathogenic mechanism underlying ischemic cardiac injury and maladaptive remodeling, offering promising therapeutic targets for ischemic heart disease. Targeting the Irgm1‐PDIA3 axis with drug therapies or using PDIA3 inhibitors like LOC14 may provide effective strategies to reduce cardiac damage post‐MI, particularly in patients with IRGM deficiency.

## 5 Experimental Section

### 5.1 Patients and Samples

A total of 58 patients with acute myocardial infarction (AMI) were recruited from the Department of Cardiology at the Second Affiliated Hospital of Harbin Medical University between April 2024 and May 2024. In short, the diagnosis of AMI was made following the universal definition [58, 59]. The diagnosis of AMI relies on an elevation of cardiac troponin I, with at least one value exceeding the 99th percentile of the reference limit, along with one or more of the following: ischemic symptoms, new ischemic electrocardiographic changes, pathological Q waves on electrocardiogram, imaging evidence of new viable myocardium loss or new regional wall motion abnormality, or coronary angiography revealing an intra‐coronary thrombus. Exclusion criteria include a history of valve replacement, cancer, recent surgery, active infection, inflammatory disease or autoimmune disease, and patients who have undergone transplantation. Additionally, 12 volunteers without a diagnosis of MI served as non‐MI controls. Exclusion criteria included a history of valve replacement, disseminated malignancy, inflammatory or autoimmune diseases, and transplant recipients. This study was approved by the Research Ethics Committee of the Second Affiliated Hospital of Harbin Medical University (GZRYS‐021 and KY2025‐266), and all participants provided informed consent. The primary characteristics of both patients and healthy controls are presented in Table S1. Blood samples were collected within 24 h of the acute event from consecutively enrolled patients with MI. Whole‐blood samples (2–3 mL) were drawn from patients with AMI and non‐MI controls into vials containing anticoagulant citrate dextrose, after which peripheral neutrophils and plasma were isolated.

### 5.2 Animals

Male C57BL/6 mice were purchased from the Beijing Vital River Laboratory Animal Technology (Beijing, China). Irgm1‐knockout‐floxed (Irgm1^flox/flox^) and S100a8‐cre mice were purchased from Cyagen Biosciences Inc. Neutrophil‐specific Irgm1‐deficient mice (Irgm1^flox/flox^S100a8‐cre, termed Irgm1‐cKO) were generated by crossing Irgm1^flox/flox^S100a8‐cre mice with S100a8‐cre mice. All mice were bred on a C57BL/6 background and maintained under pathogen‐free conditions at the Second Affiliated Hospital of Harbin Medical University. The mice were kept under 12‐hour light/dark cycles at a consistent temperature and humidity. The mice were also given ad libitum access to food and water, with no more than five animals per cage. All experimental procedures were approved by the Second Affiliated Hospital of Harbin Medical University (KY2021‐200‐02 and YJSDW2023‐155) and were performed following the ARRIVE guidelines (Animal Research: Reporting of In Vivo Experiments) for the care and use of laboratory animals.

### 5.3 MI Model

Adult 8‐12‐week‐old male mice (according to previous studies in the cardiovascular field, since the interference of protective factors such as estrogen secretion in adult female mice might mask the role of the target gene, only male adult mice were used for the study [60, 61].) were randomly divided into different groups after numbering and then were anesthetized by intraperitoneal administration of 2,2,2‐tribromoethanol (200 mg/kg) and placed on mechanical ventilation, then the left anterior descending (LAD) coronary artery was permanently occluded using a 7‐0 polypropylene suture, and the occlusion of LAD was verified by the rapid myocardial bleaching of the anterior wall of the left ventricle. The chest was then closed with a 5‐0 suture. In sham‐operated mice, the ligation was at a similar location but was not tied. Animals that died from the surgical procedure were excluded from both groups in the survival analyses. The mice were administered freshly with LOC14 (20 mg/kg, HY‐100432, MCE) daily for 7 days after the operation via oral gavage.

### 5.4 Echocardiography Analysis

After surgery, cardiac function was measured by echocardiography using a VisualSonics Vevo 3100LT ultrasound system (VisualSonics, Inc.) and a 30 MHz MX400 probe transducer at specific time points. Briefly, mice were gently anesthetized with 2,2,2‐tribromoethanol (200 mg/kg) (Aladdin, 75‐80‐9), sustaining their heart rate (HR) at 450 to 550 b.p.m. The cardiac images were obtained in two‐dimensional mode (B‐mode) in the parasternal long‐axis section. Within this section, an M‐mode cursor was placed vertically to the interventricular septum and the posterior wall of the left ventricle, at the level of the root of the papillary muscles.

### 5.5 Histology Analysis

For detecting myocardial fibrosis, the 5 µm paraffin‐embedded sections were subjected to Masson's trichrome staining (Solarbio, G1340). The infarct size of the myocardium was analyzed by triphenyltetrazolium chloride (TTC) (Solarbio, G3005). The areas of myocardial fibrosis and infarction were measured by the ImageJ software. For observing immune cell infiltration, the 5 µm paraffin‐embedded sections were subjected to H&E staining (Solarbio, G1120).

### 5.6 Immunostaining

The tissue blocks were sectioned at 5 µm for subsequent histological studies. In brief, the paraffin sections were dewaxed in xylene, rehydrated in a series of ethanols, and then washed in PBS. For antigen retrieval, the sections were rinsed in preheated citric acid buffer (pH 6.0) and boiled in a microwave for 6–10 min. After washing in PBS, the sections were blocked with 5% bovine serum albumin (BSA) and 0.5% saponin (Beyotine, P0095) for 45 min, and then incubated overnight with primary antibodies.

For immunofluorescence staining, the sections were incubated with corresponding fluorescence‐labeled secondary antibodies at room temperature for 1 h, and counterstained nuclei with DAPI (Beyotime, C1005). Apoptotic cardiomyocytes (ACs) in infarcted myocardium were assessed using the TUNEL assay (40307ES20, YEASEN). Stained tissue sections were viewed using a laser scanning confocal microscope (Zeiss).

For histochemical staining with diaminobenzidine (DAB), the sections were processed with the horseradish peroxidase (HRP)/fab polymer conjugated detection system (ZSGB, PV6001) and DAB Substrates (ZSGB, ZLI9018) according to the manufacturer's instructions, and counterstained with Mayer's hematoxylin. Stained tissue sections were viewed using a Slide Scanning System (XUYAtech, SQS‐12P).

### 5.7 In Vitro Efferocytosis Assay

WT and Irgm1‐cKO neutrophils were stimulated with LPS for 14 h, with 10^6^ 5(6)‐carboxyfluorescein succinimidyl ester (CFSE)‐labeled apoptotic/death neutrophils added for the final 1 h [62]. BMDMs were incubated with CFSE‐labeled apoptotic neutrophils for 1 h. After washing in PBS, cells were fixed with 4% PFA and labeled with anti‐CD68 mAb and their corresponding secondary antibodies, and then counterstained nuclei with DAPI. Confocal Dishes were viewed using a Laser Scanning Confocal Microscope (Zeiss). Efferocytosis index was defined as the number of BMDMs that phagocytized apoptotic neutrophils to the number of BMDMs.

### 5.8 Culture of Bone Marrow‐Derived Macrophages (BMDMs)

BMDMs were isolated and differentiated into mature macrophages as previously described from the femurs of adult mice. Briefly, bone marrow cells were flushed out from mouse femurs using RPMI 1640 and cultured in a complete RPMI 1640 medium (added with 10% FBS and 1% penicillin/streptomycin) supplemented with 25 ng/mL M‐CSF (MCE, HY‐P7085) for 7 days to generate mature BMDMs. BMDMs (2 × 10^5^ cells/mL) were seeded into 6‐well culture plates or confocal dishes.

### 5.9 Isolation of Mouse Neutrophils From Bone Marrow and Blood

The isolation of mouse neutrophils from bone marrow and blood was performed by using mouse neutrophil separation medium (TBD, Tianjin) according to the manufacturer's protocol. Isolated neutrophils from bone marrow were resuspended in RPMI 1640 for further use. Neutrophils were stimulated with 2 µg/mL LPS (coli 055:B5) for 16 or 24 h for subsequent experiment [63, 64].

For neutrophil purification, we employed the magnetic bead sorting method. In summary, following the protocol of the Mouse Neutrophil Cell Isolation Kit (BeaverBeads, 7097), neutrophils with a purity exceeding 95% could be obtained.

To test the potential relationship between the Irgm1‐PDIA3 axis and NF‐κB/caspase3 pathway, neutrophils were incubated with R‐Irgm1 (20 µg/mL, CUSABIO), Raptinal (5 µm, MCE), LOC14 (10 µm, MCE), 4‐PBA (5 mm, MCE), and Z‐DEVD‐FMK (15 µm, MCE) for 16 h.

### 5.10 Isolation of Human Neutrophils From Blood

The isolation of human blood neutrophils was performed by using a human neutrophil separation medium (TBD, Tianjin) according to the manufacturer's protocol. Briefly, a pipette was used to carefully absorb the blood sample, and the sample was added to the liquid surface of the separation solution and centrifuged for 20–30 min at 550–650 g. A pipette was used to carefully absorb the neutrophil layer in the separation solution. The plasma was prepared by centrifugation at 1000 g for 15 min at room temperature and stored at −80°C for experiments.

### 5.11 Adoptively Transferred Neutrophils

The in vitro purified WT and Irgm1‐cKO neutrophils were labeled with 5(6)‐carboxyfluorescein diacetate succinimidyl esters (CFSE, 2 µm). The cell density was maintained at 10 million/mL and stained with CFSE for 10 min at 4°C, and then they were washed twice with PBS. The labelled cells (a total of 5 × 106 cells/recipient mouse) were injected into the tail vein of recipient MI mice. After 24 h, the cells were analyzed by FACS to determine the ratio of CFSE‐labeled neutrophils in the cardiac.

### 5.12 Cell Transfection

HL‐60 cell line (CSTR:19375.09.3101HUMTCHu23) was purchased from the National Collection of Authenticated Cell Cultures (Shanghai, China). The si‐IRGM, si‐ATG5, IRGM, His‐IRGM, or Flag‐PDIA3 (Aoheng, Harbin, China) was transfected into HL‐60 cells or HEK293T cells using Lipofectamine 3000 reagent (L3000015, Invitrogen) according to the instruction manual.

### 5.13 RNA Isolation and Q‐PCR

Total RNA was isolated using the TRIzol reagent and reverse‐transcribed into complementary DNA using the ReverTra Ace qPCR RT kit (FSQ‐101, TOYOBO) according to the manufacturer's instructions. The cDNA obtained was mixed with SYBR RT‐PCR Master Mix (Q711‐02, Vozyme) and specific Q‐PCR primers (KEY RESOURCES TABLE) for Q‐PCR analysis on an ABI Prism Q6 system. The mRNA expression levels detected in each sample were normalized to β‐actin levels.

### 5.14 Protein Extraction, Immunoblot, and Immunoprecipitation Assay

Whole‐cell lysates were extracted using cell lysis buffer (Cell Signaling Technology) supplemented with a protease inhibitor cocktail and PMSF. Tissues were lysed by T‐PER buffer (ThermoFisher), added with the recommended dose of protease inhibitor cocktail, and PMSF. The concentration of the extracted protein was measured by a BCA protein assay kit (ThermoFisher). For IP assays, anti‐Irgm1 and anti‐PDIA3 antibodies were used. Immunoblot analyses were performed with anti‐Irgm1, anti‐PDIA3, anti‐caspase3, anti‐cleaved‐caspase3, anti‐GRP78, anti‐P65, an‐tiphosphorylated p65 protein, anti‐ATF6, anti‐His, anti‐β‐Actin, and anti‐GAPDH antibodies. The dilution doses of antibodies were according to the instructions. For immunoprecipitation, 1 mg protein was incubated with the indicated antibodies or IgG overnight at 4°C with constant rotation and then incubated with Protein A/G magnetic beads (Cat: B23202; Bimake; Shanghai, China) for 12 h at 4°C. Beads were washed three times with lysis buffer and resuspended in 1 × SDS‐PAGE loading buffer. Samples from immunoprecipitation or cell lysates were used for western blot analysis.

### 5.15 LC‐MS/MS Analysis

Irgm1 antibody was added to a sample of LPS‐stimulated neutrophil lysate for the IP experiment. Then, the LC‐MS/MS analysis was carried out by LUMINGBIO (Shanghai, China). Finally, we screened out the substrate proteins that could bind to Irgm1 according to the score and the mass of detected proteins. Finally, candidate proteins potentially interacting with Irgm1 were identified based on unique peptides, peptide information, relative abundance, co‐immunoprecipitation (CO‐IP) results, and other metrics.

### 5.16 Bulk RNA‐Seq Studies

Neutrophils were collected 14 h after LPS treatments. Extract RNA with TRIzol reagent. The quality was evaluated, and the qualified samples were used to construct the libraries and make the sequence. OE Biotechnology Co., Ltd (Shanghai, China) was responsible for transcriptome sequencing and analysis. *P*‐value < 0.05 and fold change > 1.5 were considered to be significantly differentially expressed. Then, performed hierarchical cluster analysis on DEGs to prove the expression patterns of genes in different groups and samples. RNA‐seq data sets were analyzed for differential expression, GO, KEGG, and GSEA.

### 5.17 ELISA Assay

Cytokine and chemokine levels were measured in mouse and human plasma by using ELISA kits according to the manufacturer's instructions (R&D Systems and Thermo Fisher Scientific).

### 5.18 Flow Cytometry Analysis

As reported previously [34], leukocytes from blood, bone marrow, and heart tissue were analyzed using flow cytometry. Blood was collected directly from the retro‐orbital vein into tubes containing 3.8% sodium citrate, followed by red blood cell lysis using RBC lysis buffer for 5 min. Cells were centrifuged at 500 g at 4°C for 5 min, and the cell pellets were resuspended in a solution containing diluted antibodies. Bone marrow samples were flushed with cold PBS using a 25‐gauge needle, passed through a 100‐µm strainer, and centrifuged at 400 g at 4°C for 3 min. Supernatants were discarded, and red blood cells were lysed with RBC lysis buffer. Dead cell staining was performed using the Zombie AquaTM Fixable Viability Kit in PBS for 15 min at 4°C. The cells were washed and resuspended in diluted antibodies before analysis. Heart tissues were incubated in a cocktail of collagenase I, hyaluronidase type I‐s, and DNase at 37°C for 1 h with gentle agitation. After digestion, single‐cell suspensions were filtered through a 70‐µm strainer into a 50 mL tube, rinsed with FACS buffer, and centrifuged at 500 g for 5 min. Dead cells were excluded from analysis by Zombie AquaTM Fixable Viability Kit staining. Cell pellets were then resuspended in diluted antibodies and incubated at room temperature for 30 min in the dark. Leukocyte subpopulations were defined as described in Figure 4, Figures S4 and S6. Antibodies against the following proteins were used: CD45‐FITC, CD11b‐APC, Ly6G‐PE, and Ly6C‐Perp‐Cy5.5 (all from BioLegend). Data were acquired using an LSRFortessa flow cytometer (BD Biosciences) and processed with FlowJo software (version 10.6.2). Myeloid leukocytes were identified as CD45^+^ and CD11b^+^ cells and further classified into Ly6G^+^ neutrophils and Ly6C^+^ monocytes [65].

### 5.19 Proximity Ligation Assay (PLA)

The PLA was performed following the NaveniFlex PLA protocol. After treatment, the cell culture on coverslips was washed with PBS three times, then fixed with 4% paraformaldehyde (PFA) for 15 min and permeabilized using 0.1% Triton X‐100 (Beyotime) for 10 min. After washing with filtered PBS, the blocking solution was added to each sample for 1 h at 37°C. The sample was incubated with the primary antibody in NaveniFlex Diluent overnight at 4°C. Subsequently, the samples were covered with a sufficient volume of Navenibody working solution and incubated in a preheated humidity chamber at 37°C for 60 min. Enzyme 1 was added to configure reaction system 1, the coverslips were covered with reaction system 1, and incubated in a preheated humidity chamber for 30 min at 37°C. Subsequently, system 2 (take care to protect from light) was configured. The coverslips were completely covered with reaction system 2 and incubated in a preheated humidity chamber for 90 min at 37°C. After washing, coverslips were counterstained with DAPI (blue) and mounted onto glass slides. Images were acquired using a Laser Scanning Confocal Microscopy (Zeiss).

### 5.20 Cell Smear Preparation and Giemsa Staining

Peripheral blood was smeared on glass slides. For Giemsa staining, cell smears were fixed in methanol for 10 min at room temperature and then stained with the Wright–Giemsa Stain Buffer (Beyotime, C0133). The nuclei stained purple.

### 5.21 Transmission Electron Microscopy (TEM)

TEM was utilized to evaluate the ultrastructural appearance of the endoplasmic reticulum (ER). LPS or LOC14‐treated neutrophils were fixed with 2.5% glutaraldehyde in 0.1 m phosphate buffer (pH = 7.4), followed by 1% osmium tetroxide (OsO4) fixation. Cells were dehydrated with a series of concentrations of ethanol, and then thin sections were stained with uranyl acetate and lead citrate for observation under a field emission scanning electron microscope.

### 5.22 Luminex Assay

Supernatants were collected after treatment as indicated. Cytokine/chemokine quantification in plasma was achieved by xMAP technology through a Luminex platform (Bio‐Rad Laboratories, Hercules, CA, USA) equipped with a magnetic washer workstation according to the manufacturer's protocol. IL‐6, Tumor Necrosis Factor‐α (TNF‐α), Interferon‐γ (IFN‐γ), and IL‐10 were analyzed.

### 5.23 Statistical Analysis

Statistical significance was analyzed by GraphPad Prism (version 9.5) with tests described in the figure legends. The number of samples was given in the individual figure legends. The results were presented as mean ± SD. The representative images were selected based on the mean value after quantitative analysis. Simple linear regression analyses were performed to examine the correlation between Irgm1 expression and other indicators. Spearman correlation analysis was used to calculate the *r* and *p* values of data distributed abnormally. The unpaired Student *t*‐test was used for comparison between 2 groups with normally distributed variables. The Mann–Whitney *U*‐test was used for comparison between 2 groups with non‐normally distributed variables. One‐way or two‐way ANOVA followed by multiple comparison tests with Bonferroni correction was used to compare differences among more than two groups. The comparisons of survival curves were performed using the Log‐rank (Mantel–Cox) test. A value of *p *< 0.05 was considered statistically significant.

## Author Contributions

S.F., X.G., Y.S., and B.Y. conceived the project. S.F. and Z.W. designed the study. Z.W., L.W., M.W., S.W., and L.X. performed the experiments. Z.W. analyzed all experiments. S.W., L.X., J.S., R.L., Y.L., J.W., F.L., and W.L. helped with experiment processing. Z.W., L.W., M.W., and J.S. participated in sample collections. W.Z. wrote the manuscript, with all authors contributing and providing feedback and advice.

## Funding

This work was supported in part by the National Natural Science Foundation of China (82370265, 62135002, and 82371930) and the Open Research Project of the Key Laboratory of Myocardial Ischemia, Ministry of Education (KF202301).

## Conflicts of Interest

The authors declare no conflict of interest.

## Supporting information




**Supporting File**: advs74228‐sup‐0001‐SuppMat.docx.

## Data Availability

The data that support the findings of this study are available from the corresponding author upon reasonable request.
