# Supplementary material for: Irgm1 Improves Postinfarction Cardiac Repair by Promoting Neutrophil Clearance and Efferocytosis
Source: Adv Sci (Weinh). 2026 Feb 25;13(21):e14863. doi: 10.1002/advs.202514863 (PMC13073322; doi:10.1002/advs.202514863)
Supplement: Supplementary file 1 — Supporting File: advs74228‐sup‐0001‐SuppMat.docx. [file ADVS-13-e14863-s001.docx]

Supporting Information

**Irgm1 Improves Postinfarction Cardiac Repair by Promoting Neutrophil Clearance and Efferocytosis**

Zeng Wang, Lai Wei, Mingyang Wang, Shanjie Wang, Lili Xiu, Jiaxiang Sun, Rongzhe Lu, Yige Liu, Jiaxin Wang, Fengyi Liu, Weike Liu, Bo Yu*, Yong Sun*, Xueqin Gao*, Shaohong Fang*

Supporting Information

**Irgm1 Improves Postinfarction Cardiac Repair by Promoting Neutrophil Clearance and Efferocytosis**

Zeng Wang, Lai Wei, Mingyang Wang, Shanjie Wang, Lili Xiu, Jiaxiang Sun, Rongzhe Lu, Yige Liu, Jiaxin Wang, Fengyi Liu, Weike Liu, Bo Yu*, Yong Sun*, Xueqin Gao*, Shaohong Fang*

**Tables**

**Table S1. Characteristics of the participants enrolled in this study.**

**
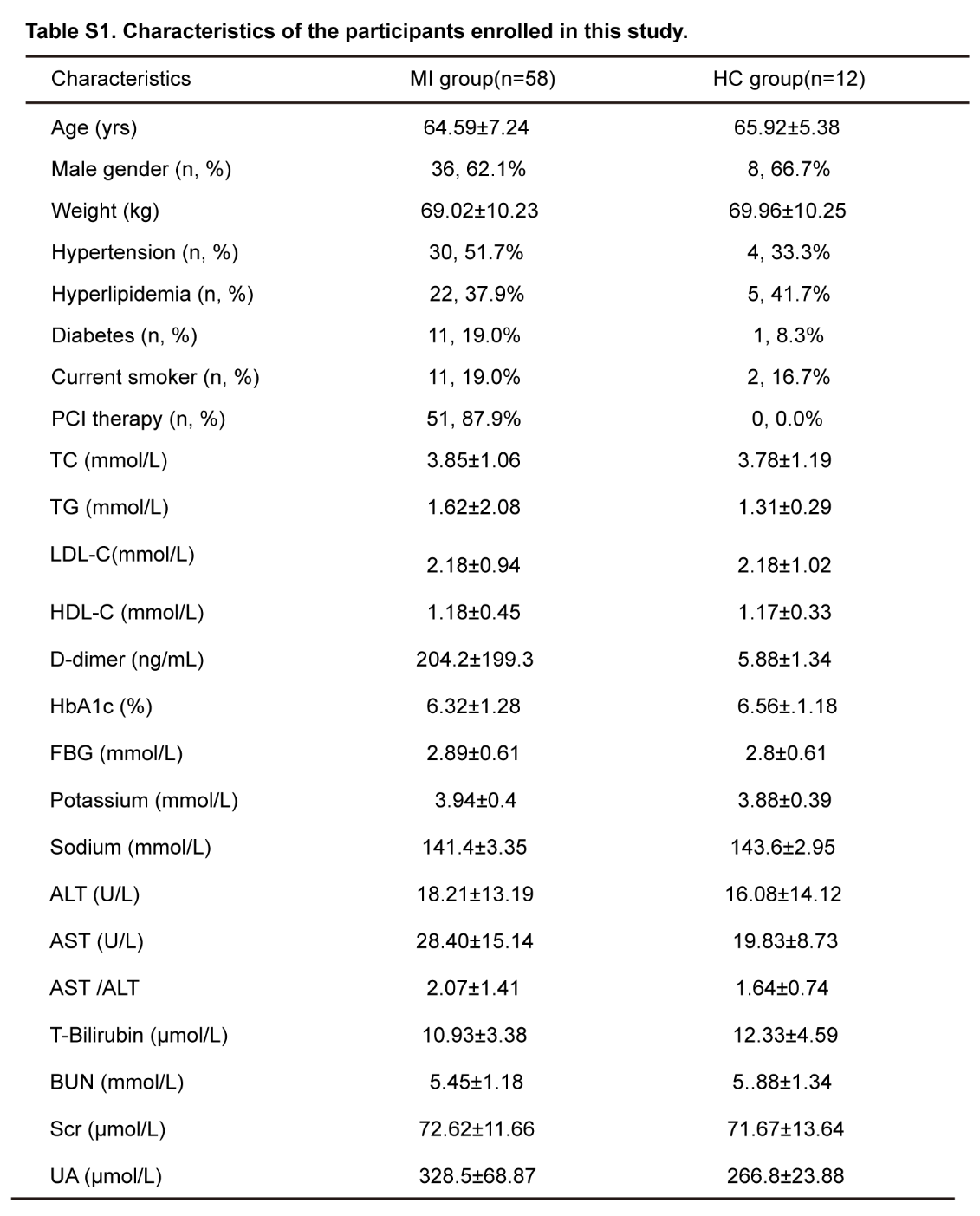
**

TC, Total Cholesterol; TG, Triglyceride; LDL-C, Low-Density Lipoprotein Cholesterol; HDL-C, High density lipoprotein cholesterol; HbA1c, glycosylated hemoglobin; FBG, fasting blood glucose; ALT, alanine aminotransferase; AST, aspartate transaminase; T-Bilirubin, total bilirubin; BUN, blood urea nitrogen; Scr, serum creatinine; UA, uric acid.

**Table S2. Characteristics of the complete blood counts.**


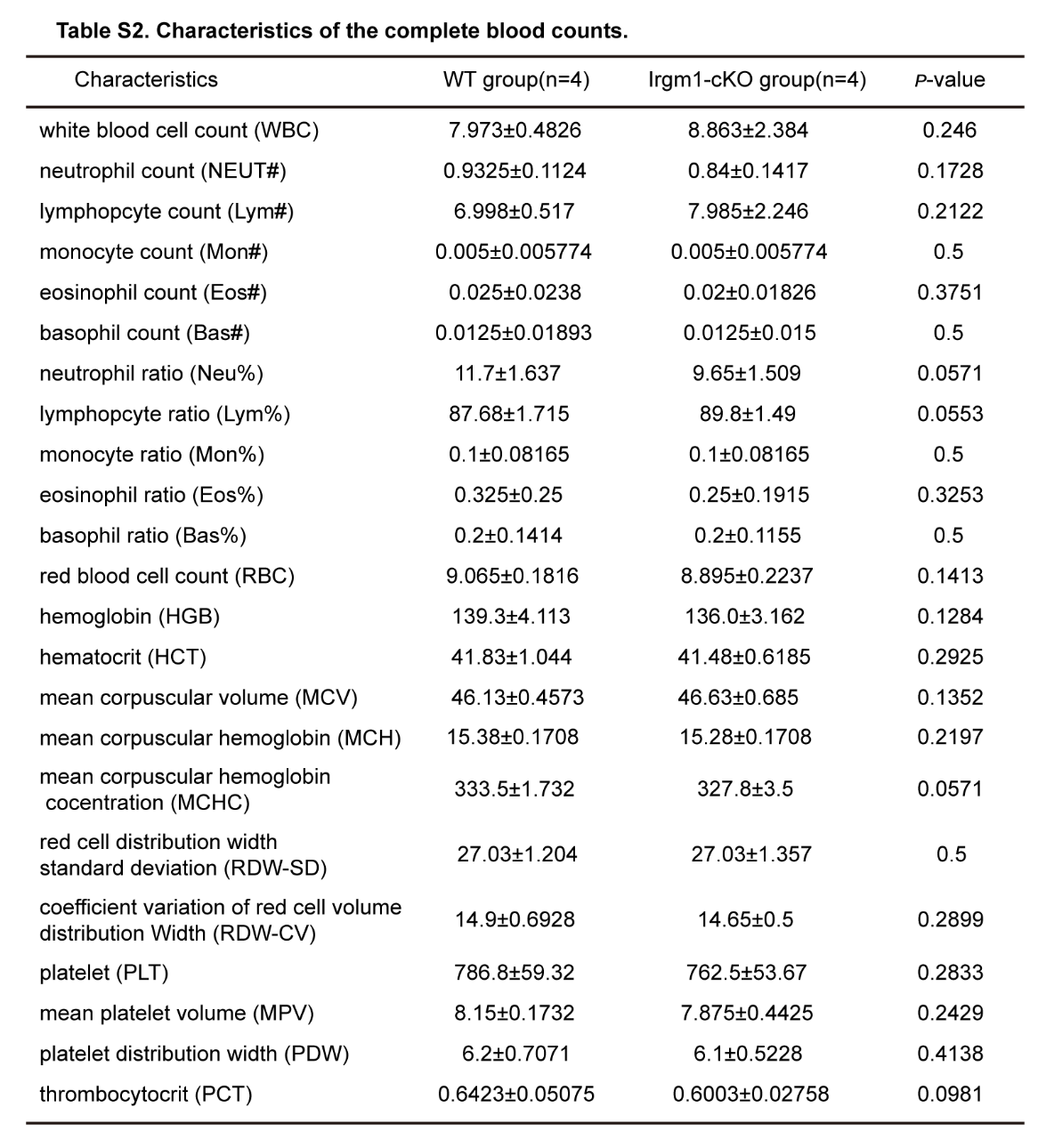


**Supplementary Figures**

**
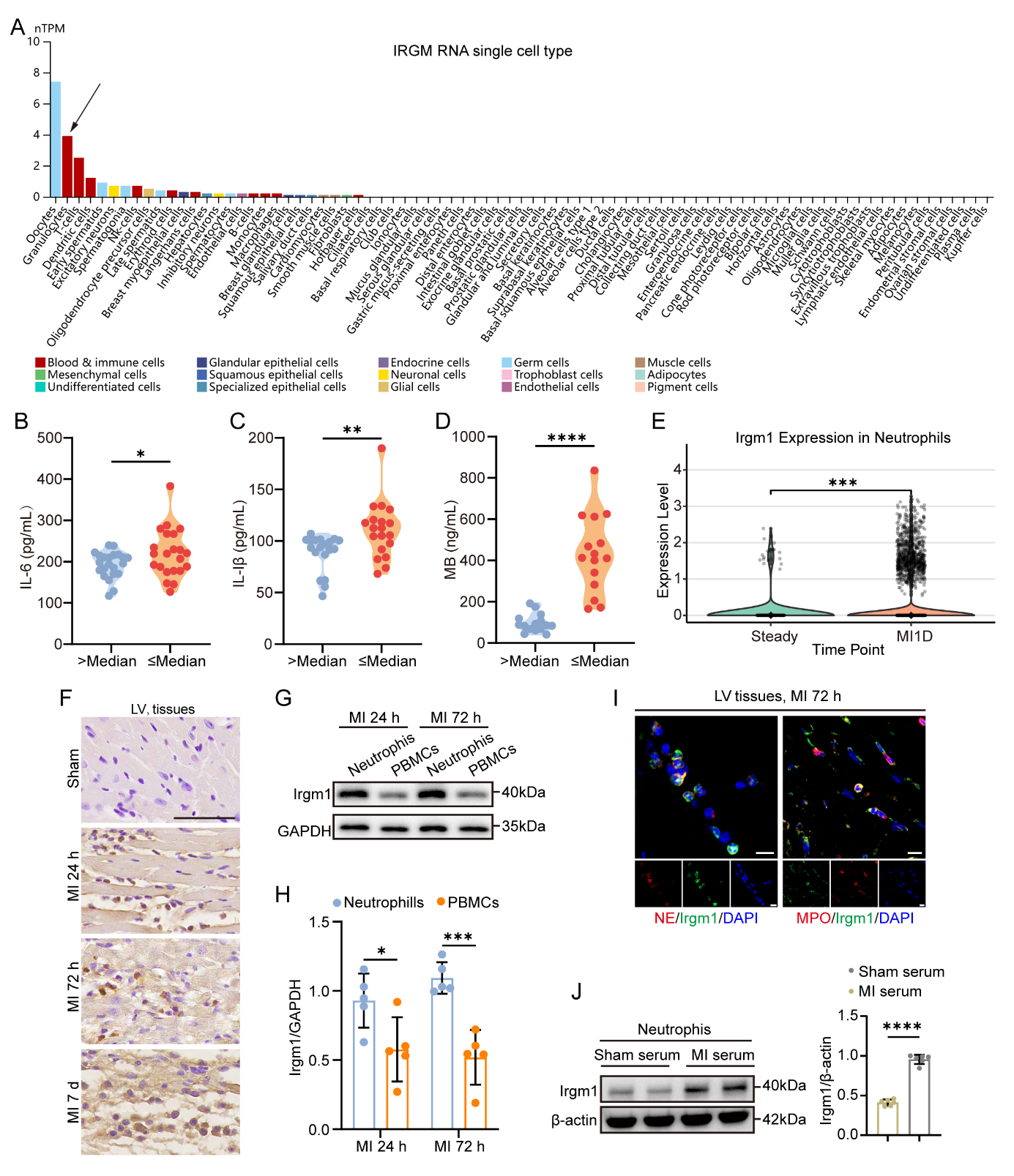
**

**Figure S1. IRGM expression level in neutrophils is related to clinical cardiac indicators in patients with MI.** (A) Expression levels of IRGM RNA across various human cell types (sourced from The Human Protein Atlas). In these cells, the highest expression is found in oocytes, followed by granulocytes, with relatively high expression in T cells, while the main cell subsets making up cardiac tissues, including cardiomyocytes, fibroblasts, endothelial cells, and smooth muscle cells, display weak IRGM expression. (B-D) The level of IL-6 (B), IL-1β (C), or MB (D) in the plasma of patients who have MI with an IRGM level greater than the median compared with patients with an IRGM level less than or equal to the median (n=22 per group for C; n=19 per group for D; n=15 per group for E; Mann-Whitney U test). (E) Expression of Irgm1 in neutrophils from baseline mice and mice at day 1 post-MI in the GSE163129 dataset. (F) Representative immunohistochemistry of Irgm1 (dark brown) in WT LV tissue at 24 h, 72 h, and 7 days post-MI with sham operation. Scale bar=50 μm. (G and H) Western blot and corresponding quantitative analysis of Irgm1 expression in peripheral blood neutrophils and PBMCs of mice after indicated times post-MI (n=5 per group; unpaired Student’s t-test). (I) Representative double immunofluorescence of Irgm1 with either NE or MPO in WT LV tissue at 72 h post-MI, showing Irgm1 expression in neutrophils. scale bar=20 μm. (J) Western blot and corresponding quantitative analysis of Irgm1 expression in neutrophils (n=6 per group; unpaired Student’s t-test). All data are means±SD. **p*<0.05, ***p*<0.01, ****p*<0.001, *****p*<0.0001.


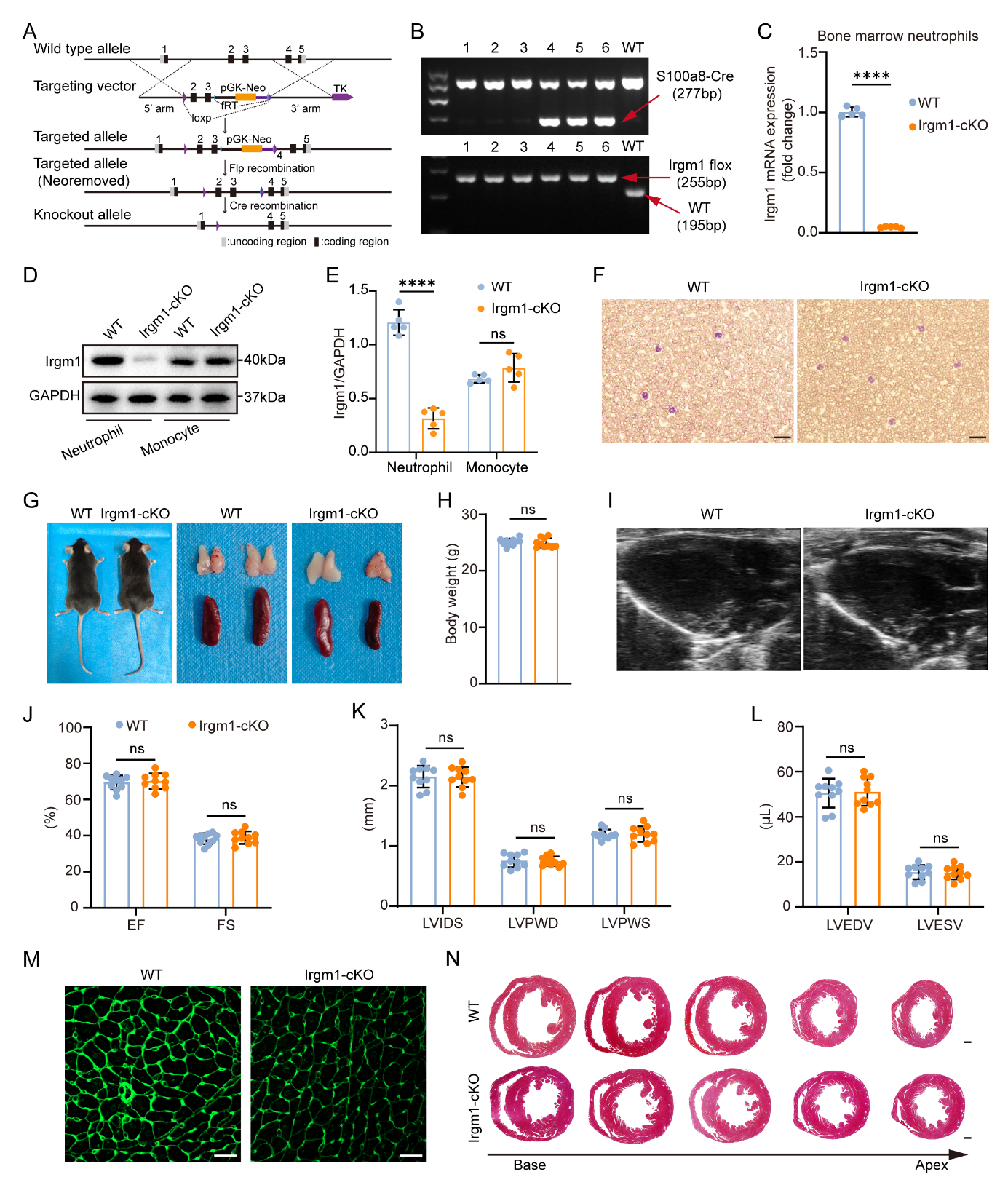


**Figure S2. Irgm1 deficiency in neutrophils does not affect cardiac function under baseline conditions.** (A) Schematic of the generation of neutrophil-specific conditional KO mice (Irgm1^flox/flox^S100a8-cre, termed Irgm1-cKO). (B) Representative PCR genotyping showing Irgm1 loxp, S100a8-cre, and WT bands in different mice. Numbers correspond to the mouse identification tags. (C) mRNA expression level of Irgm1 in neutrophils of WT and Irgm1-cKO mice (n=5 per group; unpaired Student’s t-test). (D and E) Western blot and corresponding quantitative analysis of Irgm1 were performed to confirm Irgm1 deletion in neutrophils (n=5 per group; unpaired Student t-test). (F) Giemsa staining of blood smears from WT and Irgm1-cKO mice. Scale bars=20 μm. (G) The appearances and sizes of bodies, thymus, and spleens from WT and Irgm1-cKO mice under normal conditions. (H) The body weights of 10-week-old WT and Irgm1-cKO mice (n=10 per group; unpaired Student’s t-test). (I-L) Representative M-mode echocardiograms (I) and measurements of LV ejection fraction (EF; J), fractional shortening (FS; J), dilated and systolic thickness of the left ventricular wall (LVIDS, LVPWD, LVPWS; K), or dilated and systolic LV volume (LVEDV, LVESV; L) in WT and Irgm1-cKO mice under baseline conditions (n=10 per group; 2-way ANOVA followed by Bonferroni test). (M) WGA staining of the CM area in Irgm1-cKO LV tissue with the sham operation. Scale bar=20 μm. (N) Masson trichrome staining of sequential heart sections from WT and Irgm1-cKO mice with the sham operation. Scale bar =500 μm. All data are means±SD. *****p*<0.0001. ns indicates not significant.


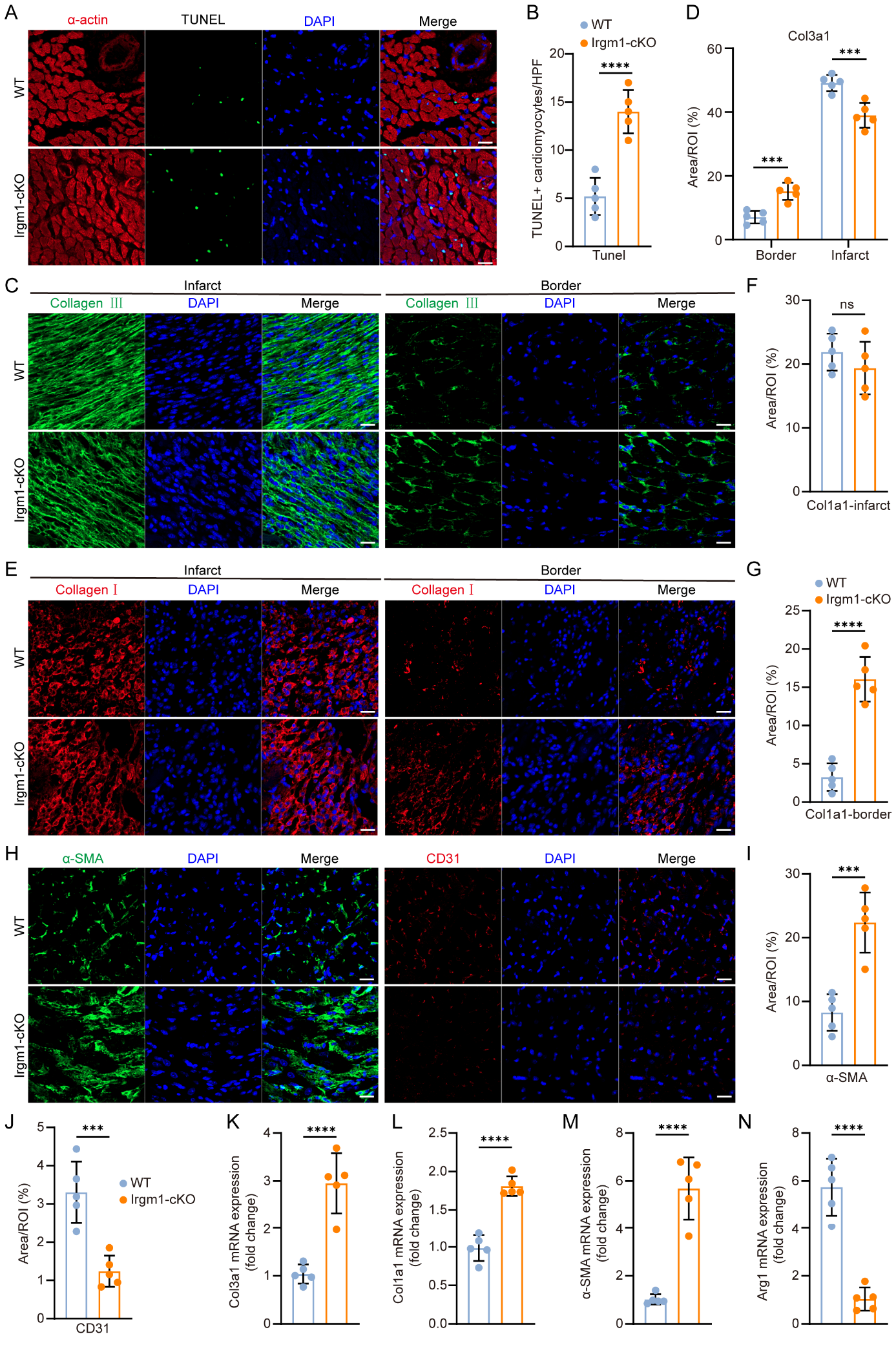


**Figure S3. Irgm1 deficiency in neutrophils suppresses cardiac repair post-MI.** (A and B) TUNEL and α-actin costaining (A) and quantification (B) of apoptotic cardiomyocytes in heart tissues from WT and Irgm1-cKO mice at day 3 post-MI (n=5 per group; unpaired Student’s t-test). Scale bar=20 μm. (C and D) Immunofluorescence staining (C) and quantification (D) of collagen III in infarct and border Zones of WT and Irgm1-cKO Mice at day 7 post-MI (n=5 per group; unpaired Student’s t-test). Scale bar=20 μm. (E-G) Immunofluorescence staining (E) and quantification (F and G) of collagen I in infarct and border Zones of WT and Irgm1-cKO mice at day 7 post-MI (n=5 per group; unpaired Student’s t-test). Scale bar=20 μm. (H-J) Immunofluorescence staining of α-SMA and CD31 in infarct tissues of WT and Irgm1-cKO mice at day 7 post-MI. Scale bar=20 μm. Quantitative data for the area/ROI of images in I and J (n=5 per group; unpaired Student’s t-test). (K-N) The mRNA expression levels of genes associated with cardiac fibrosis and myocardial repair in ischemic heart tissues of WT and Irgm1-cKO mice at day 28 post-MI (n=5 per group; unpaired Student’s t-test). All data are means±SD. ****p*<0.001, *****p*<0.0001. ns indicates not significant.


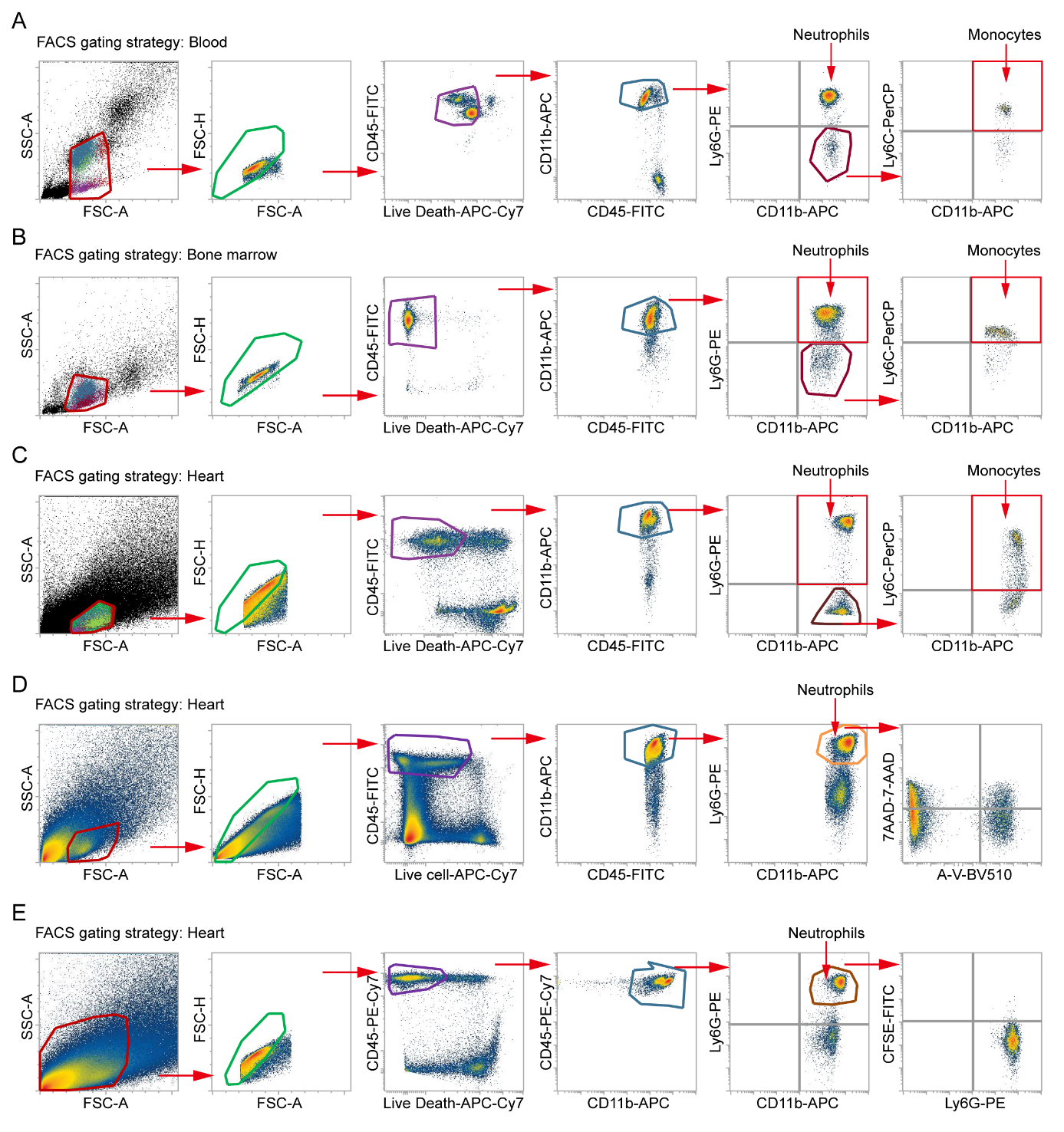


**Figure S4. Flow cytometry gating strategy of immune cells from heart tissue, in blood, and cardiac.** (A and B) Gating strategy for the identification of leukocytes in mouse blood and BM. Red blood cell-lysed blood and BM samples were stained with a fixable live-dead dye, followed by cell surface and intracellular staining. Once the doublets (by FSC-H vs. FSC-A) and dead cells were excluded, monocytes were identified as CD45^+^ CD11b^+^ and further classified as Ly6G^-^ Ly6C^+^ and Ly6G^-^ Ly6C^-^; neutrophils as CD45^+^ CD11b^+^ Ly6G^+^. (C-E) Gating strategy for identification of leukocytes in the mouse heart. RBC-free single cell suspensions were prepared from tissue digests and incubated with fixable live-dead cell dye followed by surface and intracellular staining. Once the doublets (by FSC-H vs.FSC-A) and dead cells (Live vs. Dead) were excluded, neutrophils were identified as CD45^+^, monocytes were identified as CD45^+^ CD11b^+,^ and further classified as Ly6G^-^ Ly6C^+^ and Ly6G^-^ Ly6C^-^; neutrophils as CD45^+^ CD11b^+^ Ly6G^+^. For apoptosis analysis, dead cells were included, and the expression of annexin 5 (A5)^+^ neutrophils was determined. For survival analysis of WT and Irgm1-cKO neutrophils, WT and Irgm1-cKO neutrophils were analyzed based on CFSE staining.


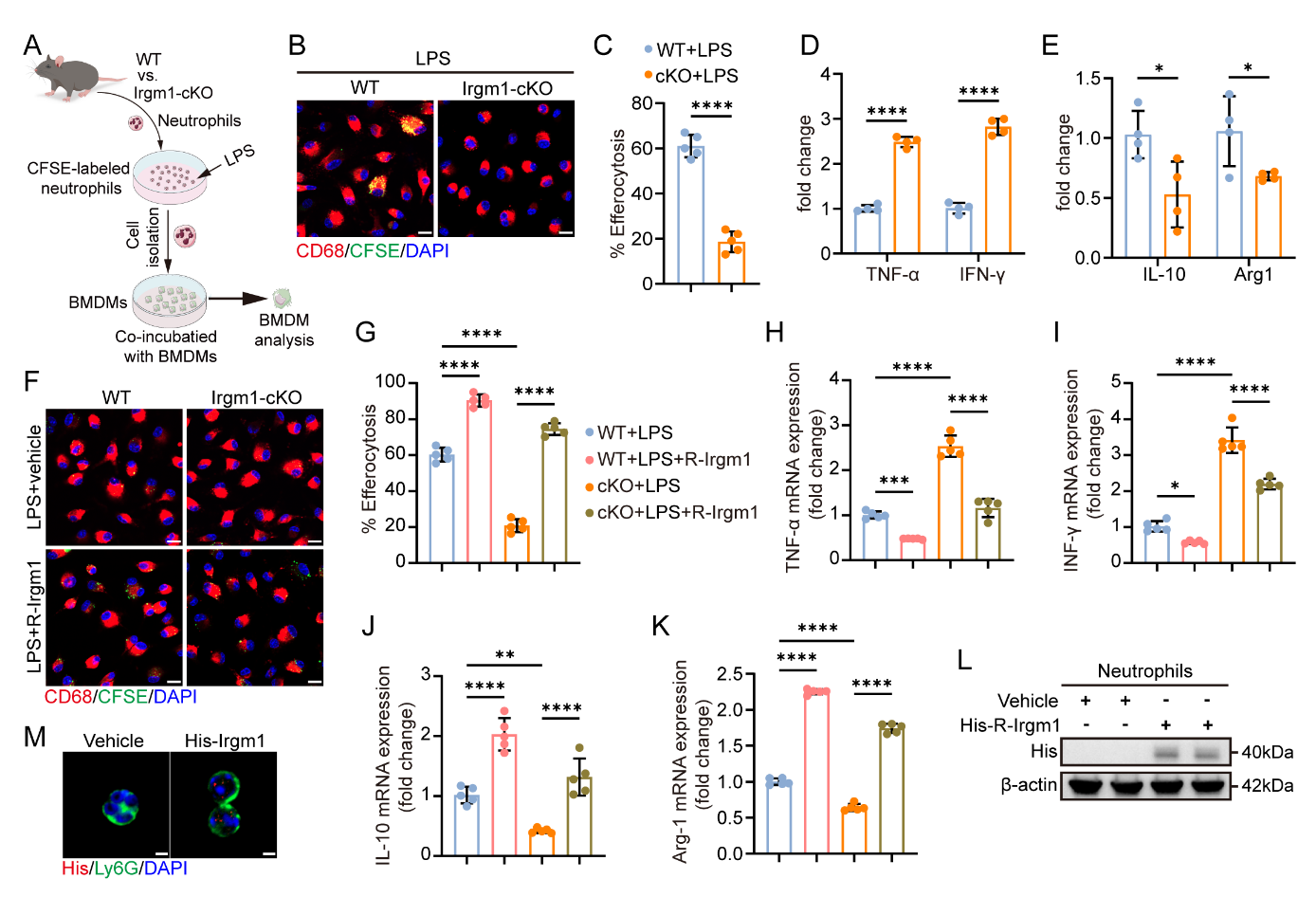


**Figure S5. Neutrophil-specific Irgm1 deficiency reduces efferocytosis and hinders the macrophage reparative phenotype.** (A) Schematic illustration of the experimental protocol for neutrophils co-cultured with macrophages. CFSE: 5(6)-carboxyfluorescein succinimidyl ester. LPS: Lipopolysaccharides. BMDMs: Bone Marrow-Derived Macrophages. (B) Immunofluorescence images showing BMDMs in the process of engulfing neutrophils. Neutrophils were labeled with CFSE after LPS stimulation. Scar bar=10 μm. (C) Efferocytosis index from B (n=5 per group; unpaired Student’s t-test). The efferocytosis index was defined as the number of BMDMs that phagocytosed neutrophils in the total number of BMDMs. (D and E) The mRNA expression levels of proinflammatory genes (D, TNF-α, and IFN-γ) and anti-inflammatory genes (E, IL-10, and Arg-1) in BMDMs phagocytizing neutrophils (n=4 per group; unpaired Student’s t-test). (F) Immunofluorescence images showing BMDMs in the process of engulfing neutrophils. Neutrophils cultured with LPS and R-Irgm1 were subsequently labeled with CFSE . Scar bar=10 μm. (G) Efferocytosis index from F (n=5 per group; one-way ANOVA followed by Bonferroni test). (H-K) The mRNA expression levels of proinflammatory genes (H, TNF-α and I, IFN-γ ) and anti-inflammatory genes (J, IL-10 and K, Arg-1) in BMDMs phagocytizing neutrophils treated with R-Irgm1 and LPS (n=5 per group; one-way ANOVA followed by Bonferroni test). (L) Western-blot analysis of His expression in neutrophils treated with or not with His-Irgm1. (M) Representative images of immunofluorescence staining of His^+^ and Ly6G in neutrophils treated with or not with His-Irgm1. All data are means±SD. **p*<0.05, ***p*<0.01, ****p*<0.001, *****p*<0.0001.

**
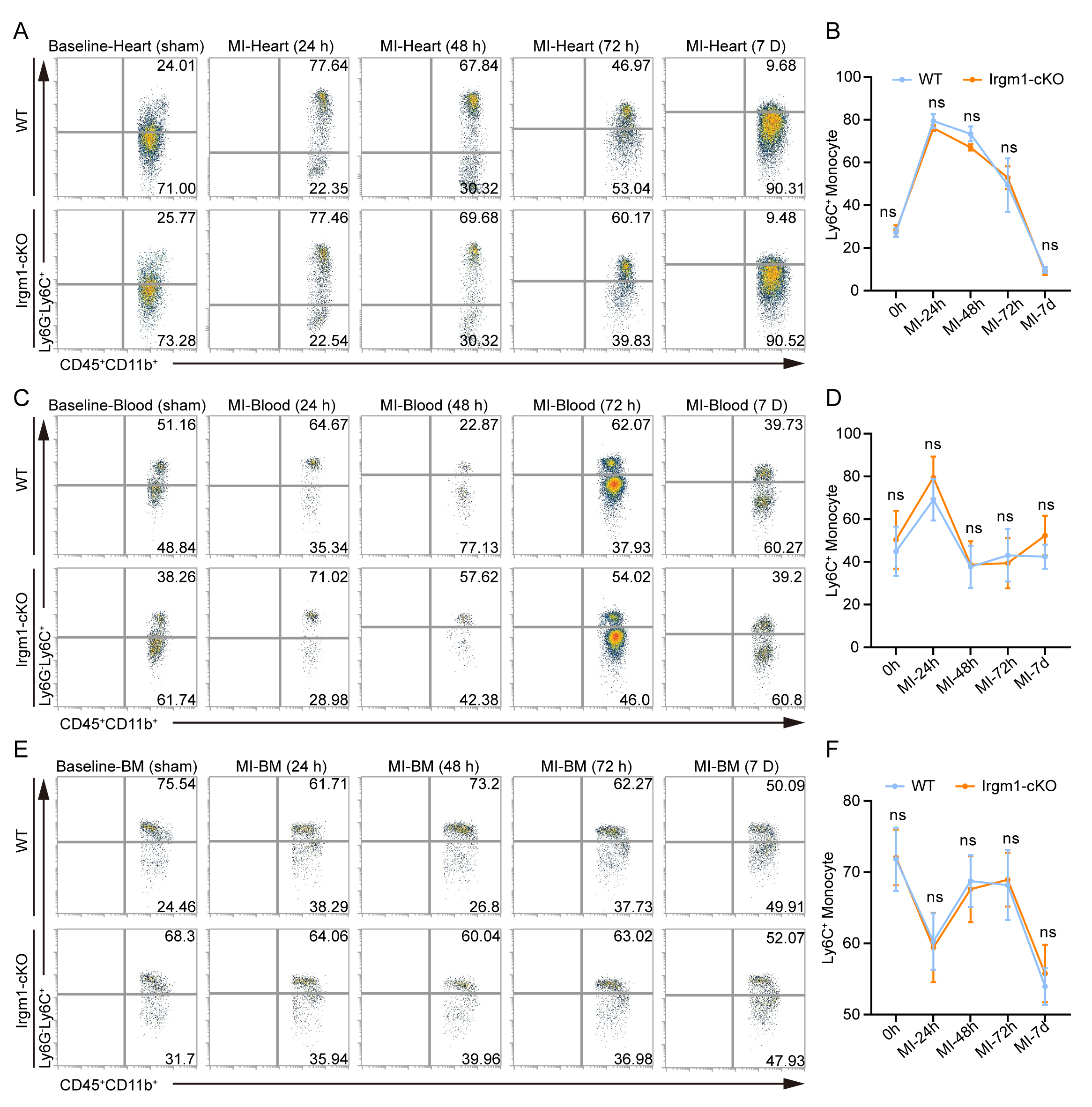
**

**Figure S6. Neutrophil-specific Irgm1 deficiency does not affect the infiltration of monocytes/macrophages post-MI.** (A-B) Flow cytometric analysis and quantification of monocytes (CD45^+^, CD11b^+^, Ly6G^-^, Ly6C^-^ or Ly6C^+^) in heart tissue from WT and Irgm1-cKO mice at different time points (24 h, 48 h, 72 h and 7 D) post-MI or sham operation, along with their quantification (B). (n=5 per group; 2-way ANOVA followed by Bonferroni test). (C-D) Flow cytometric analysis and quantification of monocytes (CD45^+^, CD11b^+^, Ly6G^-^, Ly6C^-^ or Ly6C^+^) in blood from WT and Irgm1-cKO mice at different time points (24 h, 48 h, 72 h and 7 Days) post-MI or sham operation, along with their quantification (D). (n=5 per group; 2-way ANOVA followed by Bonferroni test). (E-F) Flow cytometric analysis and quantification of monocytes (CD45^+^, CD11b^+^, Ly6C^+^) in bone marrow (BM) from WT and Irgm1-cKO mice at different time points (24 h, 48 h, 72 h and 7 Days) post-MI or sham operation, along with their quantification (F). (n=5 per group; 2-way ANOVA followed by Bonferroni test). All data are means±SD. ns indicates not significant.


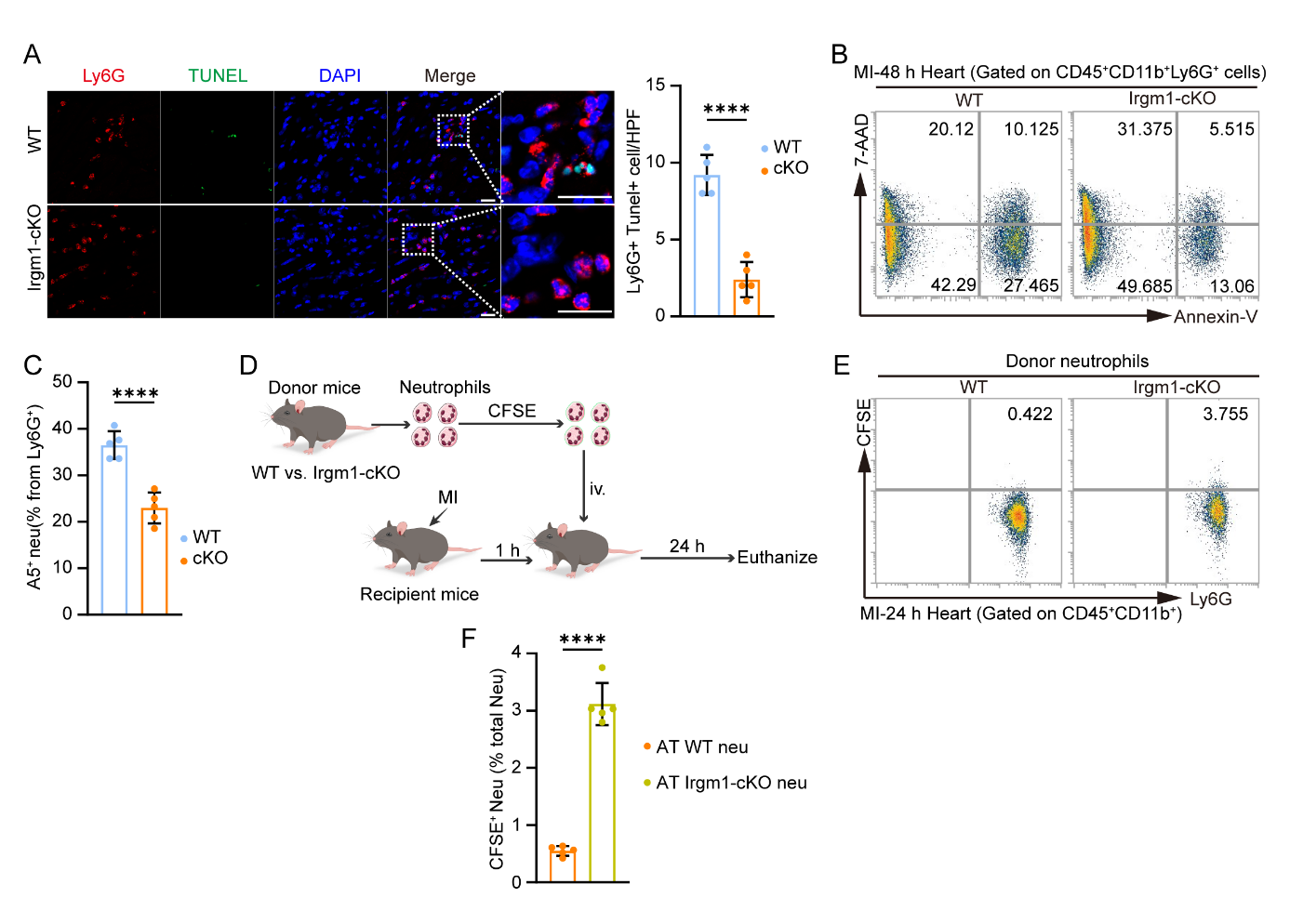


**Figure S7. Neutrophil-specific Irgm1 deficiency inhibits neutrophil apoptosis and prolongs neutrophil lifespan.** (A) TUNEL and Ly6G co-localization staining and quantitative analysis of apoptotic neutrophils in the infarct area of WT and Irgm1-cKO mice on day 3 post-MI. (n=5 per group, unpaired Student’s t-test). Scale bar=20 μm. (B) Flow cytometric analysis of apoptotic neutrophils stained with annexin 5 (A5) and 7-AAD in heart tissues of WT and Irgm1-cKO mice at day 2 post-MI. (C) Quantification of the percentages of apoptotic neutrophils in total Ly6G^+^ cells (n=5 per group, unpaired Student’s t-test). (D) Schematic diagram of adoptively transferred neutrophils in the MI model. (E) Flow cytometric analysis of adoptively transferred neutrophils stained with CFSE in heart tissues at 24 h post-MI. (F) Quantification of the percentages of adoptively transferred neutrophils in total Ly6G^+^ cells at 24 h post-MI (n=5 per group; unpaired Student’s t-test). All data are means±SD. *****p*<0.0001.


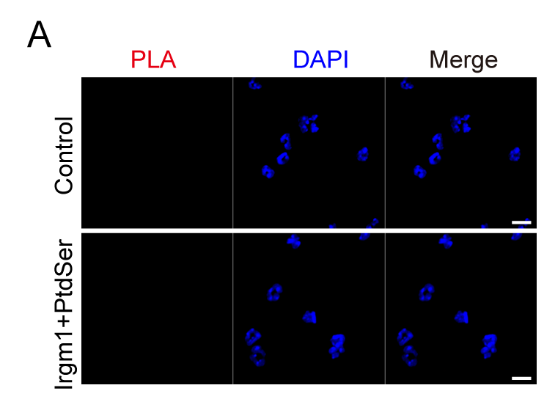


**Figure S8. Irgm1 was not bound directly to PtdSer.** Representative PLA staining image assessing the direct interaction between Irgm1 and PtdSer. Scale bar=10 μm.


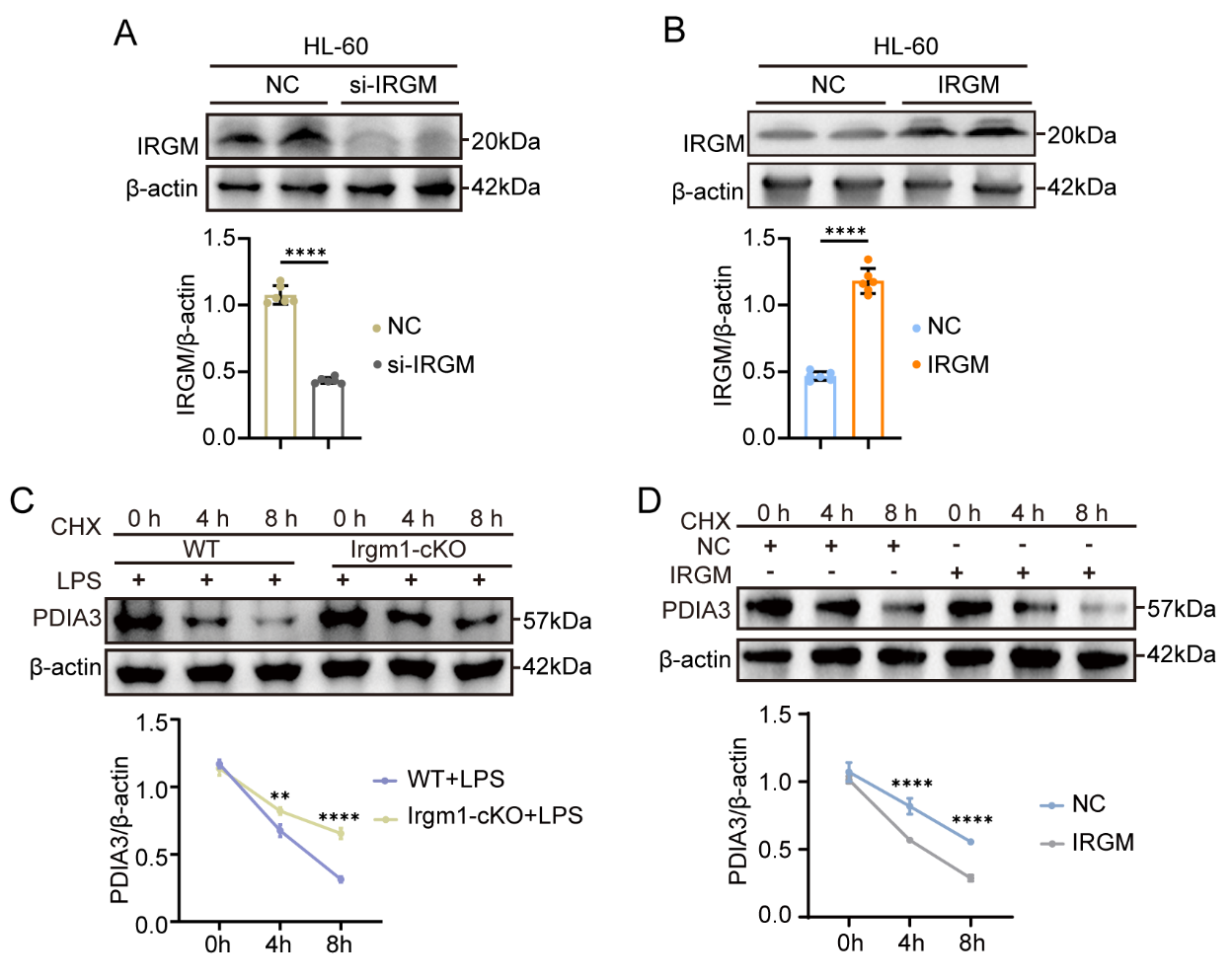


**Figure S9. The half-life of PDIA3 following Irgm1 deletion and IRGM overexpression**

(A) Efficiency of si-IRGM in HL-60 cells by Western blot assay (n=6 per group; unpaired Student’s t-test). (B) Efficiency of IRGM overexpression plasmid in HL-60 cells by Western blot assay (n=6 per group; unpaired Student’s t-test). (C) The half-life of PDIA3 was prolonged in Irgm1-cKO neutrophil (n=3 per group; two-way ANOVA followed by Bonferroni test). (D) The half-life of DIA3 was shortened with overexpression of IRGM in HL-60 cells (n=3 per group; two-way ANOVA followed by Bonferroni test). Data are represented as the mean ± SD. **p* <0.05; *****p* < 0.0001.

**
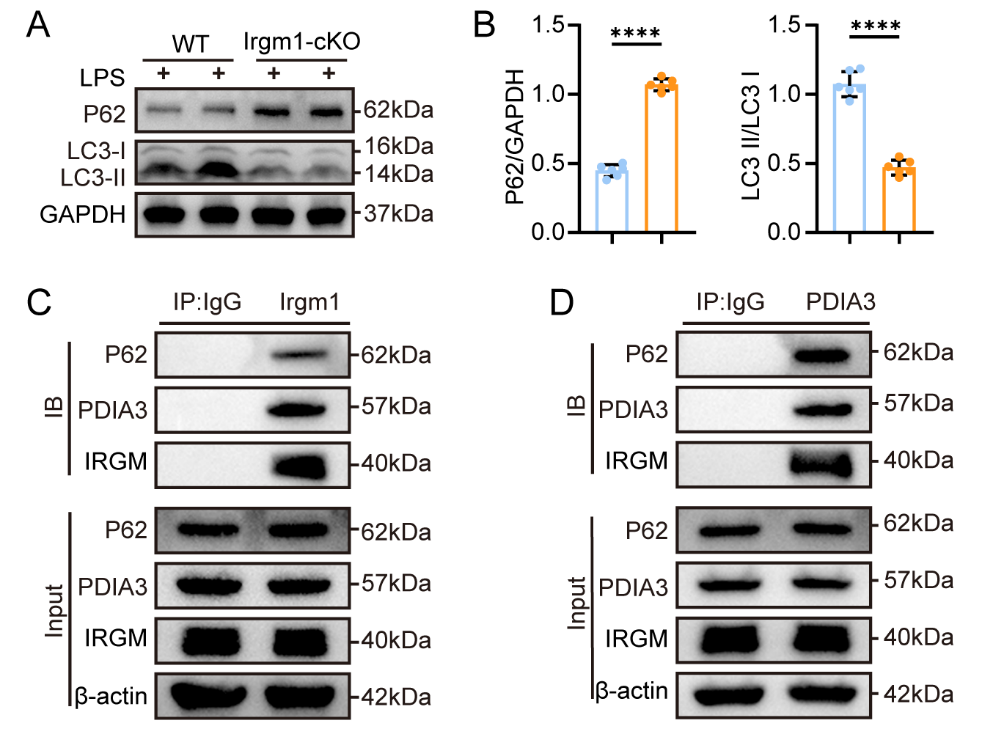
**

**Figure S10. Irgm1 deficiency inhibits neutrophil autophagy.**

(A-B) Representative bands (A) and (B) quantification of proteins P62 and LC3II/I in neutrophils treated with LPS from WT and Irgm1-cKO mice (n=6 per group; unpaired Student’s t-test). (C) Western blot analysis of P62 and PDIA3 immunoprecipitated by Irgm1. (D) W Western blot analysis of Irgm1 and P62 immunoprecipitated by PDIA3. All data are means±SD. *****p*<0.0001.

**
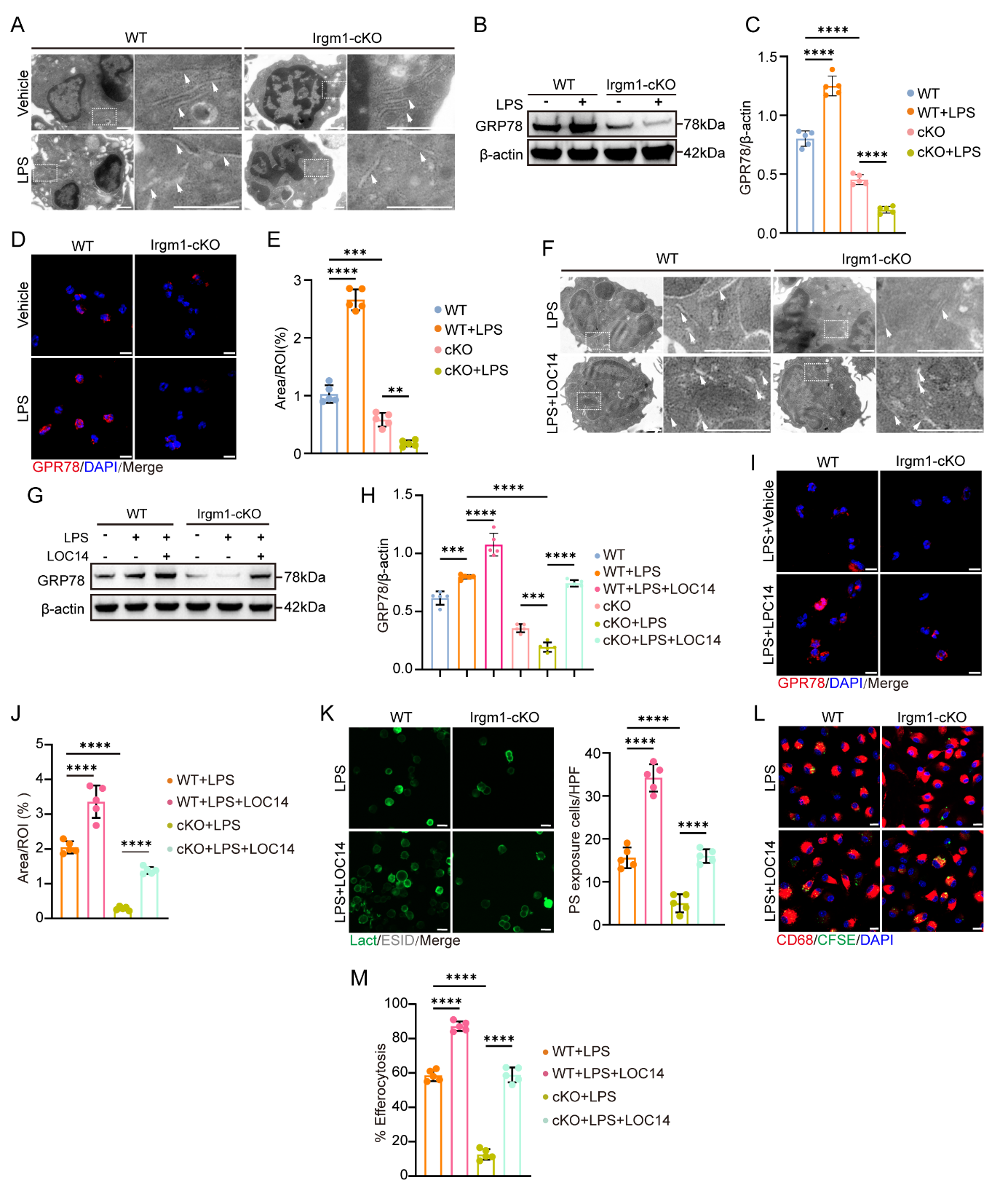
**

**Figure S11. The Irgm1-PDIA3 axis regulates neutrophil clearance and efferocytosis via ER Stress.** (A) TEM images of WT and Irgm1-cKO neutrophils incubated with LPS. White arrows indicate ER. Scale bars=1 μm. (B and C) Western-blot analysis (B) and quantification (C) of Irgm1 expression in neutrophils treated with LPS (n=5 per group; one-way ANOVA followed by Bonferroni test). (D and E) GRP78 expression (D) and semiquantitative analysis (E) by confocal detection (n=5 per group; one-way ANOVA followed by Bonferroni test). Scale bar=20 μm. (F) TEM images of WT and Irgm1-cKO neutrophils incubated with LPS and LOC14. White arrows indicate ER. Scale bars=1 μm. (G and H) Western-blot analysis (G) and quantification (H) of Irgm1 expression in neutrophils treated with LPS and LOC14 (n=5 per group; one-way ANOVA followed by Bonferroni test). (I and J) GPR78 expression (I) and semiquantitative analysis (J) by confocal detection (n=5 per group; one-way ANOVA followed by Bonferroni test). Scale bar=20 μm. (K) FITC-conjugated lactadherin (FITC-Lact) staining of Ptdser exposure in neutrophils with LPS and LOC14 treatment and its quantitative analysis (n=5 per group; one-way ANOVA followed by Bonferroni test). Scale bar=10 μm. (L and M) Immunofluorescence images showing BMDMs in the process of engulfing neutrophils (L) and their efferocytosis index (M) (n=5 per group; one-way ANOVA followed by Bonferroni test). Scale bar=10 μm. Label neutrophils with CFSE after treatment with LPS and LOC14. All data are means±SD. **p*<0.05, ***p*<0.01, ****p*<0.001, *****p*<0.0001.


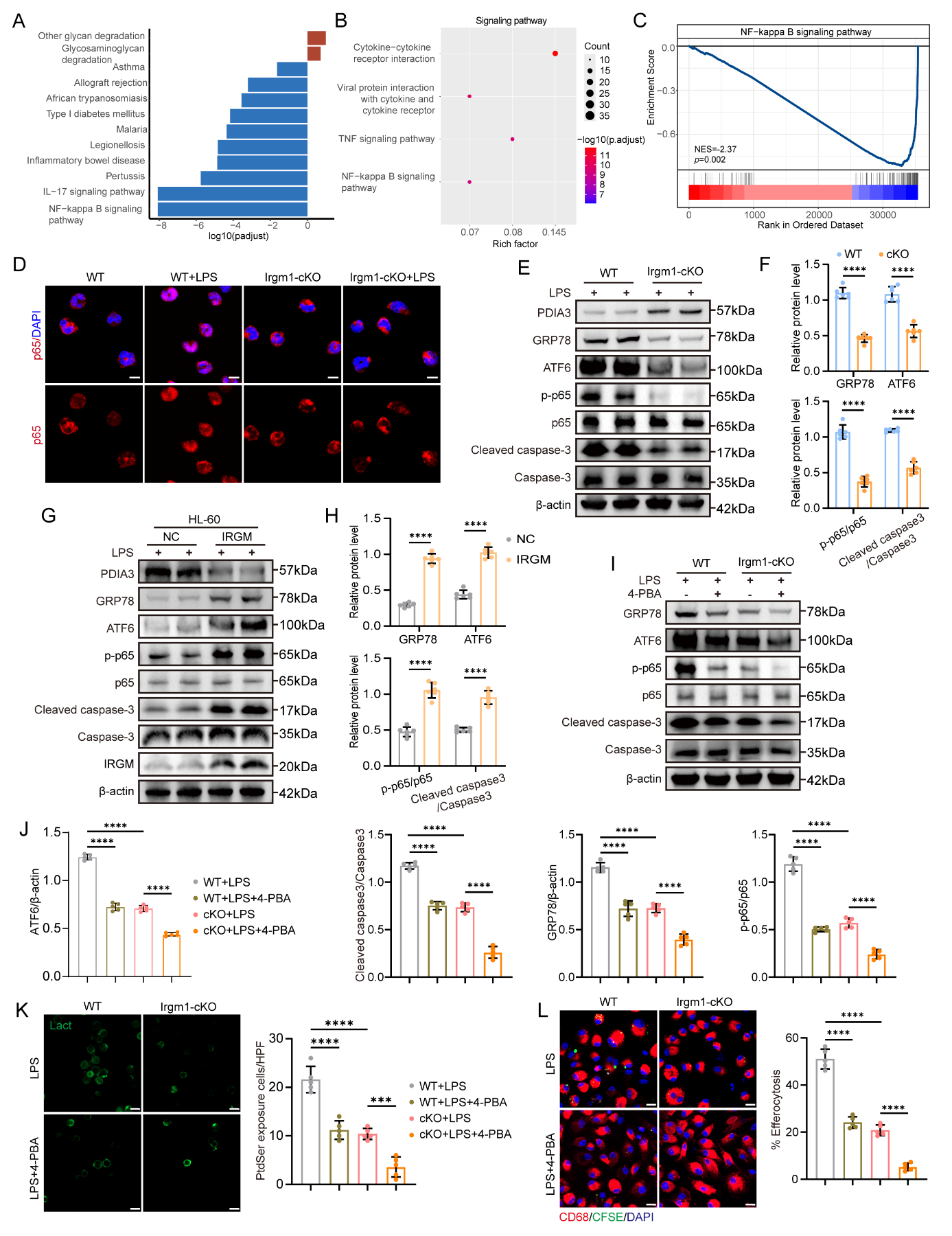


**Figure S12. The Irgm1-PDIA3 axis regulates neutrophil clearance and efferocytosis through the NF-κB/caspase-3 pathway.** (A) Gene ontology (GO) analysis showing key molecular signatures regulated by Irgm1 in WT and Irgm1-cKO neutrophils after LPS stimulation. (B) The differentially expressed genes (DEGs) in WT and Irgm1-cKO neutrophils were subjected to KEGG analysis for differential signaling pathways after LPS stimulation. (C) Gene Set Enrichment Analysis (GSEA) showing the enrichment score of NF-κB signaling pathway gene sets in the WT and Irgm1-cKO neutrophils after LPS stimulation. (D) The confocal images of WT and Irgm1-cKO neutrophils immunostained with p65 antibody after treatment with LPS. Scale bar=5 μm. (E-F) Representative bands (E) and (F) quantification of proteins GRP78, ATF6, p-65 and cleaved caspase3 in neutrophils treated by LPS (n=6 per group; unpaired Student’s t-test). (G-H) Representative bands (G) and (H) quantification of proteins GRP78, ATF6, p-65 and cleaved caspase3 in neutrophils treated by IRGM plsmid (n=6 per group; unpaired Student’s t-test). (I-J) Representative bands (I) and (J) quantification of proteins GRP78, ATF6, p-65 and cleaved caspase3 in neutrophils treated by LPS and 4-PBA (n=5 per group; one-way ANOVA followed by Bonferroni test). (K) FITC-conjugated lactadherin (FITC-Lact) staining of Ptdser exposure in neutrophils with LPS and 4-PBA treatment and its quantitative analysis (n=5 per group; one-way ANOVA followed by Bonferroni test). Scale bar=10 μm. (L) Immunofluorescence images showing BMDMs in the process of engulfing neutrophils and their efferocytosis index (n=5 per group; one-way ANOVA followed by Bonferroni test). Scale bar=10 μm.

All data are means±SD. **p*<0.05, ***p*<0.01, ****p*<0.001, *****p*<0.0001.


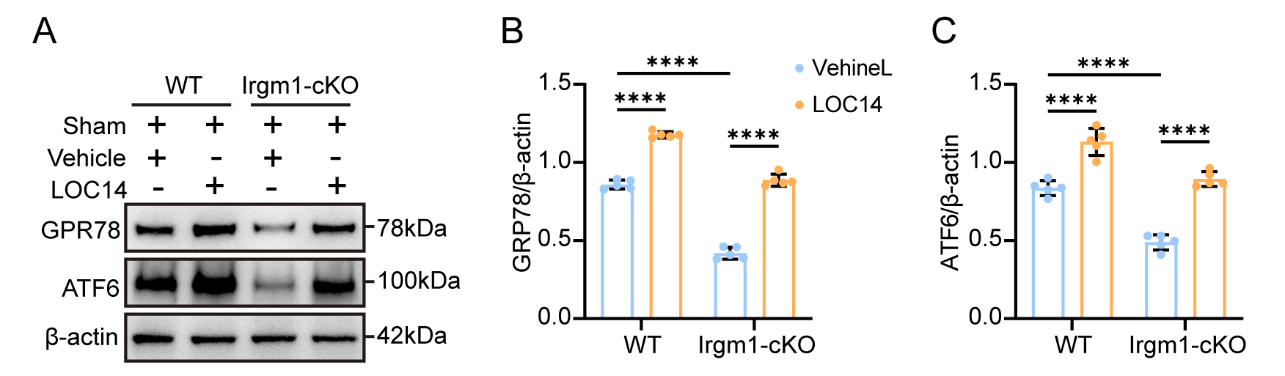


**Figure S13. Administration of LOC14 improves ER stress inhibition caused by Irgm1 deficiency in vivo.**

(A-C) Representative bands (A) and ( B and C) quantification of proteins GRP78 and ATF6 in neutrophils from WT and Irgm1-cKO mice at day 3 post-MI (n=5 per group; two-way ANOVA followed by Bonferroni test). All data are means±SD. *****p*<0.0001.

**
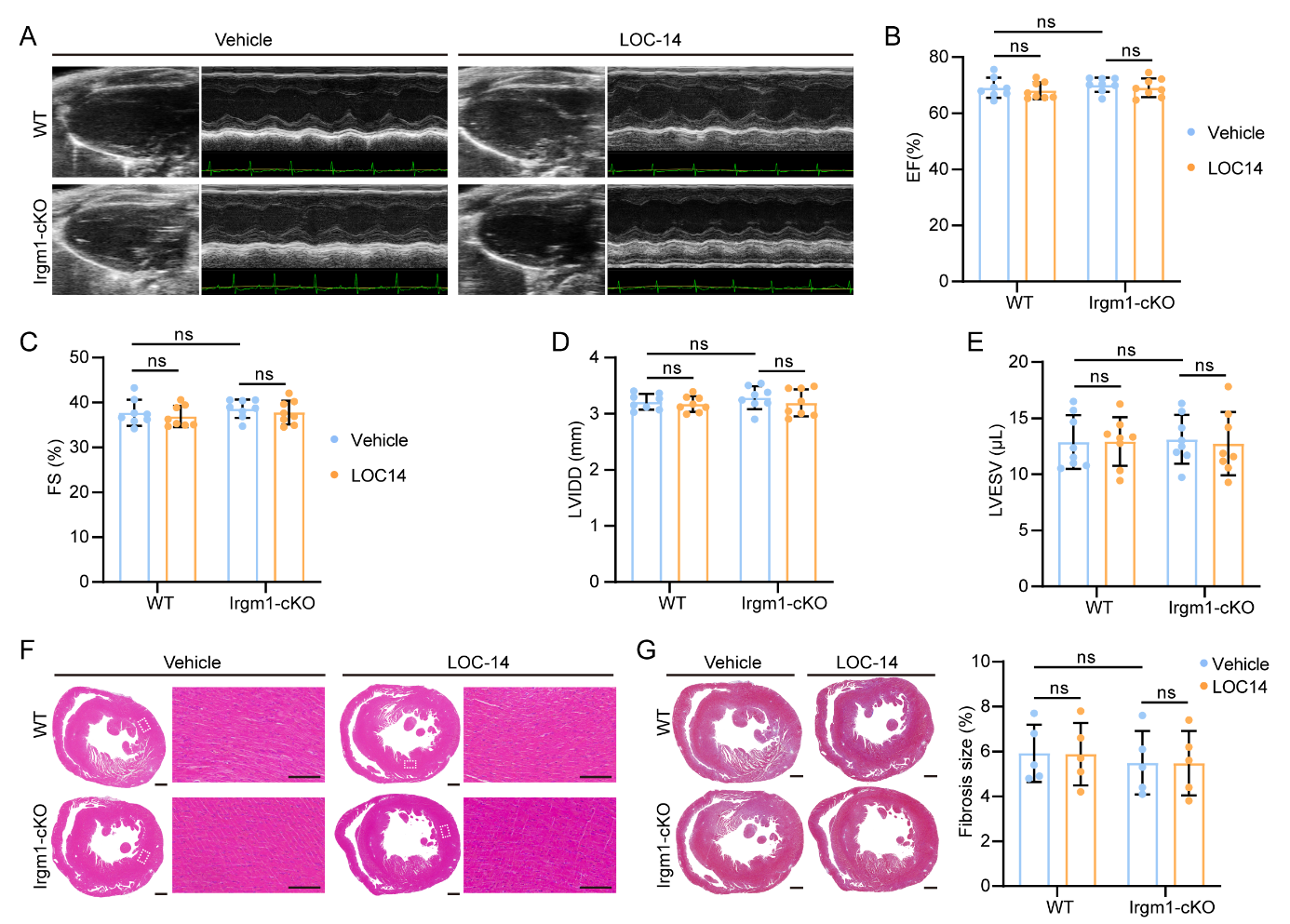
Figure S14. Administration of LOC14 has no therapeutic effect on WT and Irgm1-cKO mice after sham operation.**

(A) Representative parasternal long-axis views and M-mode echocardiogram images obtained from WT and Irgm1-cKO mice given Vehicle or LOC14 at day 14 after sham operation. (B-E) Echocardiographic analyses of ejection fraction (EF; B), fractional shortening (FS; C), left ventricular internal diameter in Diastole (LVIDD; D), and left ventricular end- systolic volume (LVESV; E) from WT and Irgm1-cKO mice given Vehicle or LOC14 at day 14 after sham operation. (n=8 per group; two-way ANOVA followed by Bonferroni test). (F) H&E staining of cardiac sections from WT and Irgm1-cKO mice given Vehicle or LOC14 at day 14 after sham operation. Scale bars: 100 μm for high magnification, 500 μm for low magnification. (G) Masson trichrome staining of sequential heart sections from WT and Irgm1-cKO mice given Vehicle or LOC14 at day 14 after sham operation and the quantified size of fibrotic areas (n=5 per group; two-way ANOVA followed by Bonferroni test). Scale bar=500 μm. All data are means±SD. ns indicates not significant.

**KEY RESOURCES TABLE**

| REAGENTS or RESOURCES | SOURCE | IDENTIFIER |
| --- | --- | --- |
| Antibodies |  |  |
| FITC-CD45+A3:C46 | Biolegend | Cat# 157214 |
| PE-cy7-CD45 | Biolegend | Cat# 103113 |
| APC-CD11b | Biolegend | Cat# 101212 |
| PE-Ly-6G | Biolegend | Cat# 127608 |
| PerCP-Ly-6C | Biolegend | Cat# 128028 |
| Annexin V | Biolegend | Cat# 640937 |
| 7-AAD | Biolegend | Cat# 420403 |
| Viability Kit | Biolegend | Cat# 423105 |
| IRGM | GeneTex | Cat# GTX85038 |
| Irgm1 | CST | Cat# ab25377 |
| Ly6G | Abcam | Cat# GTX00669 |
| Ly6G | Servicebio | Cat# GB11229-50 |
| MPO | R&D Systems | Cat# AF3667 |
| NE | Abcam | Cat# ab310335 |
| ATF6 | Proteintech | Cat# 24169-1-AP |
| CD31 | Abcam | Cat# ab9498 |
| LC3B | Abcam | Cat# ab192890 |
| P62 | Proteintech | Cat# 18420-1-AP |
| α-SMA | CST | Cat# 48938 |
| α-actin | Sigma | Cat# A7811 |
| CD68 | Servicebio | Cat# GB153150-50 |
| cTnI | Proteintech | Cat# 66376-1-Ig |
| CD68 | Abcam | Cat# ab125212 |
| Collagen Type I | Proteintech | Cat# 67288-1-Ig |
| Collagen Type III | Proteintech | Cat# 22734-1-AP |
| His | Proteintech | Cat# 66005-1-Ig |
| ERp57/ERp60 | Proteintech | Cat# 15967-1-AP |
| ERp57/ERp60 | Proteintech | Cat# 66423-1-Ig |
| GRP78/BIP | Proteintech | Cat# 66574-1-Ig |
| NF-κB p65 | Proteintech | Cat# 80979-1-RR |
| Phospho-NF-κB p65 | CST | Cat# 3033 |
| WGA | Sigma(L4895) | N/A |
| Caspase-3 | CST | Cat# 9662 |
| Cleaved Caspase-3 | CST | Cat# 9661 |
| PtdSer | Sigma | Cat# 05-719 |
| β-actin | ZSGB-BIO | Cat# TA-09 |
| GAPDH | ZSGB-BIO | Cat# TA-08 |
| Goat Anti-Mouse IgG | ZSGB-BIO | Cat# ZB-2305 |
| Goat Anti-rabbit IgG | ZSGB-BIO | Cat# ZB-2301 |
| Goat Anti-Rabbit (AF594) | Abcam | Cat# ab150080 |
| Goat Anti-Mouse (AF647) | Abcam | Cat# ab150115 |
| Goat Anti-Mouse (AF488) | CST | Cat# 4408 |
| ***Continued*** |  |  |
| REAGENTS or RESOURCES | SOURCE | IDENTIFIER |
| Goat Anti-Rat (AF488) | Abcam | Cat# ab150157 |
| Goat Anti-Rabbit (AF647) | CST | Cat# 4414 |
| Goat Anti-Rabbit (AF488) | CST | Cat# 4412 |
| Goat Anti-Rat (AF647) | CST | Cat# 4418 |
| Goat Anti-Mouse (AF594) | CST | Cat# 8890 |
| Goat Anti-Rat (AF594) | Abcam | Cat# ab150160 |
| Donkey Anti-Goat (AF488) | Abcam | Cat# ab150129 |
| Donkey Anti-Goat (AF594) | Abcam | Cat# ab150132 |
| Donkey Anti-Goat (AF488) | Abcam | Cat# ab150135 |
| Biological Samples | | |
| Human plasma | The Second Affiliated Hospital of Harbin Medical University | N/A |
| Chemicals，peptides，and recombinant proteins | | |
| 2,2,2-tribromoethanol | Aladdin | Cat# 75-80-9 |
| Masson’s Trichrome Stain Kit | Solarbio | Cat# G1340 |
| TTC Solution | Solarbio | Cat# G3005 |
| Hematoxylin-Eosin(HE) Stain Kit | Solarbio | Cat# G1120 |
| 0.5 % saponin | Beyotine | Cat# P0095 |
| DAPI | Beyotine | Cat# C1005 |
| TUNEL Apoptosis Detection Kit | YEASEN | Cat# 40307ES20 |
| the horseradish peroxidase (HRP)/Fab Polymer Conjugated Detection System | ZSGB | Cat# PV6001 |
| DAB Substrates | ZSGB | Cat# ZLI9018 |
| MCSF | MCE | Cat# HY-P7085 |
| mice neutrophils separation medium | TBD | Cat# LZS1100 |
| human neutrophils separation medium | TBD | Cat# LZS11131 |
| Raptinal | MCE | Cat# HY-121320 |
| Z-DEVD-FMK | MCE | Cat# HY-12466 |
| LOC14 | MCE | HY-100432 |
| ReverTra Ace qPCR RT kit | TOYOBO | Cat# FSQ-101 |
| 4-PBA | MCE | Cat# HY-A0281 |
| Diprovocim | MCE | Cat# HY-123942 |
| ***Continued*** |  |  |
| REAGENTS or RESOURCES | SOURCE | IDENTIFIER |
| NaveniFlex Cell MR RED | Navinci | Cat# NC.MR.100.Red |
| ChamQ Universal SYBR qPCR Master Mix | Vozyme | Cat# Q711-02 |
| RIPA lysis buffer | Beyotime | Cat# P0013D |
| TrueBlack Lipofuscin Autofluorescence Quencher | Biotium | Cat# 23007 |
| PMSF | Beyotime | Cat# ST505 |
| BCA protein assay kit | Thermo Fisher | Cat# 23225 |
| SDS-PAGE loading buffer | Beyotime | Cat# P0015L |
| Wright-Giemsa Stain Buffer | Beyotime | Cat# C0133 |
| RPMI-1640 | Thermo Fisher | Cat# C11875500CP |
| 10% FBS | ScienCell | Cat# 0500 |
| LPS | Biotopped | Cat# L2880D |
| CFSE | Absin | Cat# 150347-59-4 |
| Tissue Storage Solution | MACS | Cat# 130-100-008 |
| Hyaluronidase | Biosharp | Cat# 37326-33-3 |
| Collagenase II | Biosharp | Cat# BS164-100mg |
| Collagenase I | Biosharp | Cat# BS163-100mg |
| DNaseI | Biosharp | Cat# BS137-10mg |
| D-PBS | Beyotime | Cat# C0221G |
| HBSS | Biotopped | Cat# TOP0049 |
| Recombinant Human IRGM His Protein | Novubio | Cat# NBP2-23131 |
| Mouse Irgm1 Recombinant Protein | CUSABIO | Cat# CSB-YP730655MO |
| Protein A/G Magnetic Beads for IP | Bimake | Cat# B23201 |
| BLAC-FITC | Prolytix | Cat# NN0427-1ML |
| Critical commercial assays | | |
| Human IRGM elisa kit | MyBioSource | Cat# MBS9320915 |
| Human CPR elisa kit | MEIMIAN | Cat# MM-0135H1 |
| Human IL-1β elisa kit | MEIMIAN | Cat# MM-0181H1 |
| Human IL-6 elisa kit | MEIMIAN | Cat# MM-0049H1 |
| Neutrophil Cell Isolation Kit | BEAVER | Cat# 70907 |
| Experimental models: Organisms/strains | | |
| Mouse: C57BL/6- S100a8-cre | Cyagen Biosciences Inc | N/A |
| Mouse: C57BL/6-Irgm1^flox/flox^ | Cyagen Biosciences Inc | N/A |
| ***Continued*** |  |  |
| REAGENTS or RESOURCES | SOURCE | IDENTIFIER |
| Mouse: Wild type (C57BL/6J) | Vital River Laboratory Animal Technology | Cat #Jackson 000664; |
| Oligonucleotides for real time PCR |  |  |
| TNF-α-F(h) | General Biosystems | 5’-CCTCTCTCTAATCAGCCCTCTG-3’ |
| TNF-α-R(h) | General Biosystems | 5’-GAGGACCTGGGAGTAGATGAG-3’ |
| IFN-γ-F(h) | General Biosystems | 5’-TCGGTAACTGACTTGAATGTCCA-3’ |
| IFN-γ-R(h) | General Biosystems | 5’-TCGCTTCCCTGTTTTAGCTGC-3’ |
| IL-10-F(h) | General Biosystems | 5’-GACTTTAAGGGTTACCTGGGTTG-3’ |
| IL-10-R(h) | General Biosystems | 5’-TCACATGCGCCTTGATGTCTG-3’ |
| Arg1-F(h) | General Biosystems | 5’-GTGGAAACTTGCATGGACAAC-3’ |
| Arg1-R(h) | General Biosystems | 5’-AATCCTGGCACATCGGGAATC-3’ |
| IRGM-F(h) | General Biosystems | 5’-GCCATGAATGTTGAGAAAGCCT-3’ |
| IRGM-R(h) | General Biosystems | 5’-GTCCTGGACACTATCTTCAGAGT-3’ |
| ACTIN-F(h) | General Biosystems | 5’-CCAGCCTTCCTTCTTGGGTAT-3’ |
| ACTIN-R(h) | General Biosystems | 5’-GGGTGAAAACGCAGCTCAG-3’ |
| IL-6-F(m) | General Biosystems | 5’-ACAAAGCCAGAGTCCTTCAGAG-3’ |
| IL-6-R(m) | General Biosystems | 5’-TTGGATGGTCTTGGTCCTTAGC-3’ |
| TNF-α-F(m) | General Biosystems | 5’-CAGGCGGTGCCTATGTCTC-3’ |
| TNF-α-R(m) | General Biosystems | 5’-CGATCACCCCGAAGTTCAGTAG-3’ |
| IFN-γ-F(m) | General Biosystems | 5’-ATGAACGCTACACACTGCATC-3’ |
| IFN-γ-R(m) | General Biosystems | 5’-CCATCCTTTTGCCAGTTCCTC-3’ |
| IL-10-F(m) | General Biosystems | 5’-CTTACTGACTGGCATGAGGATCA-3’ |
| IL-10-R(m) | General Biosystems | 5’-GCAGCTCTAGGAGCATGTGG-3’ |
| Arg1-F(m) | General Biosystems | 5’-CTCCAAGCCAAAGTCCTTAGAG-3’ |
| Arg1-R(m) | General Biosystems | 5’-GGAGCTGTCATTAGGGACATCA-3’ |
| α-SMA-F(m) | General Biosystems | 5’-ACCGTGGAGAGTTTCGTGG-3’ |
| α-SMA-R(m) | General Biosystems | 5’-GCTGGCTGGATACCTGAAGC-3’ |
| Col1a1-F(m) | General Biosystems | 5’-TAAGGGTCCCCAATGGTGAGA-3’ |
| Col1a1-R(m) | General Biosystems | 5’-GGGTCCCTCGACTCCTACAT-3’ |
| Col3a1-F(m) | General Biosystems | 5’-CCTGGCTCAAATGGCTCAC-3’ |
| Col3a1-R(m) | General Biosystems | 5’-GACCTCGTGTTCCGGGTAT-3’ |
| Irgm1-F(m) | General Biosystems | 5’-TGGCAATGGCATGTCATCTT-3’ |
| Irgm1-R(m) | General Biosystems | 5’-AGTACTCAGTCCGCGTCTTCGT-3’ |
| ***Continued*** |  |  |
| REAGENTS or RESOURCES | SOURCE | IDENTIFIER |
| ACTIN-F(m) | General Biosystems | 5’-GGCTGTATTCCCCTCCATCG-3’ |
| ACTIN-R(m) | General Biosystems | 5’-CCAGTTGGTAACAATGCCATGT-3’ |
| Software and algorithms | | |
| ImageJ | NIH | <https://imagej.net/ij/> |
| Prism | GraphPad Software | [https://www.graphpad.com](https://www.graphpad.com/scientificsoftware/prism/) |
|  |  | [/scientificsoftware/prism/](https://www.graphpad.com/scientificsoftware/prism/) |
| Flowjo | BD | <https://www.flowjo.com/> |
| Adobe Illustrator | Adobe | <https://www.adobe.com/cn/> |
| Adobe Photoshop | Adobe | <https://www.adobe.com/cn/> |
| R v4.4.1 | N/A | <https://www.r-project.org/> |
| Rstudio | N/A | <https://rstudio.com/> |
| ZEN3 | ZEISS | [https://www.zeiss.com.cn](https://www.zeiss.com.cn/microscopy/l/campaigns/) |
|  |  | [/microscopy/l/campaigns/](https://www.zeiss.com.cn/microscopy/l/campaigns/) |
